# Distance-Based Functional Diversity Measures and Their Decomposition: A Framework Based on Hill Numbers

**DOI:** 10.1371/journal.pone.0100014

**Published:** 2014-07-07

**Authors:** Chun-Huo Chiu, Anne Chao

**Affiliations:** Institute of Statistics, National Tsing Hua University, Hsin-Chu, Taiwan; Institute of Botany, Czech Academy of Sciences, Czech Republic

## Abstract

Hill numbers (or the “effective number of species”) are increasingly used to characterize species diversity of an assemblage. This work extends Hill numbers to incorporate species pairwise functional distances calculated from species traits. We derive a parametric class of functional Hill numbers, which quantify “the effective number of equally abundant and (functionally) equally distinct species” in an assemblage. We also propose a class of mean functional diversity (per species), which quantifies the effective sum of functional distances between a fixed species to all other species. The product of the functional Hill number and the mean functional diversity thus quantifies the (total) functional diversity, i.e., the effective total distance between species of the assemblage. The three measures (functional Hill numbers, mean functional diversity and total functional diversity) quantify different aspects of species trait space, and all are based on species abundance and species pairwise functional distances. When all species are equally distinct, our functional Hill numbers reduce to ordinary Hill numbers. When species abundances are not considered or species are equally abundant, our total functional diversity reduces to the sum of all pairwise distances between species of an assemblage. The functional Hill numbers and the mean functional diversity both satisfy a replication principle, implying the total functional diversity satisfies a quadratic replication principle. When there are multiple assemblages defined by the investigator, each of the three measures of the pooled assemblage (gamma) can be multiplicatively decomposed into alpha and beta components, and the two components are independent. The resulting beta component measures pure functional differentiation among assemblages and can be further transformed to obtain several classes of normalized functional similarity (or differentiation) measures, including *N*-assemblage functional generalizations of the classic Jaccard, Sørensen, Horn and Morisita-Horn similarity indices. The proposed measures are applied to artificial and real data for illustration.

## Introduction

Functional diversity quantifies the diversity of species traits in biological communities, and is widely regarded as a key to understanding ecosystem processes and environmental stress or disturbance [1]–[11]. A higher functional diversity signifies greater differences among species trait values, more distinct ecological functions, and thus potentially better functional stability to perturbations caused by human impacts or environment stresses [12]–[15]. Thus, it is critical to quantify functional diversity properly [16].

Functional diversity is typically quantified by using measures based on species trait values and species abundance (or any measure of species importance, e.g., cover or biomass). A wide array of functional diversity measures have been developed in the literature [4], [17]–[22]; see [23]–[26] for reviews. There are three major approaches to construct functional diversity measures: trait-value-based [27], dendrogram-based [4], [28]–[31], and distance-based [17], [32]–[35]. For the trait-value-based approach, measures are calculated from species trait values directly. In the dendrogram-based approach, a functional dendrogram is constructed by applying a clustering algorithm to the species pairwise distance matrix. However, it has been shown that different clustering methods may lead to different conclusions [21], [30], [31]. An unavoidable issue in the dendrogram-based approach is how to select a clustering algorithm to construct a functional dendrogram.

This paper is focused on the distance-based approach which does not require a dendrogram. The selection of clustering algorithm can thus be avoided. A commonly used functional diversity index in the distance-based approach is *FAD* (Functional Attribute Diversity), the sum of pairwise distances between species [17]. However, *FAD* does not take into account species abundances. Rao's quadratic entropy *Q* and its transformations have also been extensively applied to quantify functional diversity [32]–[35]. The measure *Q*, a generalization of the traditional Gini-Simpson index, incorporates both species pairwise distances and species abundances. However, it inherits mathematical properties of the Gini-Simpson index which are inappropriate for a diversity measure [35], [36]–[38]. The problems with interpreting *Q* as a diversity measure will be briefly discussed and illustrated by examples later in this paper. Ricotta and Szeidl [35] and de Bello et al. [39] resolved these problems by converting *Q* to “species equivalents”; see later text for details. However, we show here that their solution in its original form does not behave properly for non-ultrametric distance matrices. (A distance metric *d* is ultrametric if it satisfies the criterion *d*(*x*, *y*)≤max{*d*(*x*, *z*), *d*(*y*, *z*)} for all *x*, *y* and *z*.) In many applications, the distance matrices calculated from species traits do not satisfy this criterion and thus are non-ultrametric. For example, the commonly used Gower distance matrices calculated from three habitats in our real data (see *Examples and Applications*) are all non-ultrametric. Also, measures based on quadratic entropy gives common species much more weight than their population fraction. It would be more informative to have a parameter to control the sensitivity of the measure to species abundances. We were thus motivated to derive a new parametric class of measures that are valid for both ultrametric and non-ultrametric matrices.

Our framework is based on Hill numbers, a one-parameter family of diversity indices (differing among themselves only by a parameter *q* which determines the sensitivity to the relative abundances) that incorporate species richness and relative abundances. Hill numbers include species richness, Shannon diversity (the exponential of entropy) and Simpson diversity (inverse of the Simpson index). They were first used in ecology by MacArthur [40], developed by Hill [41], and recently reintroduced to ecologists by Jost [42], [43]. A very brief description of Hill numbers is provided below.

Hill numbers are increasingly used to characterize abundance-based species diversity of an assemblage; see a series of papers in a recent forum [44]. An important advantage of using Hill numbers is that Hill numbers obey an intuitive *replication principle*, an essential mathematical property that captures biologists' intuitive notion of diversity [40], [41]; see *Conclusion and Discussion* for more details. The replication principle requires that if we have *N* equally diverse, equally large assemblages with no species in common, the diversity of the pooled assemblage must be *N* times the diversity of a single assemblage. We refer to the special case of *N* = 2 as a “doubling property” as defined in [36]. Hill numbers were recently extended to incorporate phylogenetic distance and dendrogram-based functional distance between species [45] while still satisfying the replication principle.

This work first generalizes Hill numbers to distance-based *functional Hill numbers*, which quantify “the effective number of equally abundant and (functionally) equally distinct species”. Throughout this paper, species are *equally distinct* if all species pairwise functional distances are a fixed constant. To fully characterize distance-based functional diversity, we also need measures in units of “distance”. The product of our functional Hill number and Rao's quadratic entropy (the abundance-weighted mean distance between species) quantifies the *mean functional diversity* (per species), i.e., the effective sum of functional distances between a fixed species to all other species (plus intraspecific distance if exists). The product of the functional Hill number and the mean functional diversity thus quantifies the *total functional diversity* (or simply *functional diversity*), the effective total distance between species of the assemblage. When all species are equally distinct, our functional Hill numbers reduce to ordinary Hill numbers. When species abundances are not considered or species are equally abundant, our total functional diversity reduces to *FAD* [17]. Thus our approach also extends *FAD* to incorporate species abundances. Different perspectives regarding the distance-based approaches and the replication principle can be found in [46], [47], [48] and [48], respectively.

When there are multiple assemblages defined by the investigator, Hill numbers can be multiplicatively partitioned into independent (or unrelated) alpha and beta components [43], [49]. An advantage of our approach is that each of the three functional diversity measures we propose (functional Hill numbers, mean functional diversity and total functional diversity) can be used for complete multiplicative partitioning. The beta component can be further transformed onto the range [0, 1] to obtain normalized measures of functional similarity (or differentiation), including *N*-assemblage functional generalizations of the classic Jaccard, Sørensen, Horn and Morisita-Horn similarity indices. Our framework thus unites functional diversity measures and functional similarity (or differentiation) among assemblages. Our previous work on Hill numbers covered taxonomic diversity and phylogenetic diversity. With the present development of functional Hill numbers, we now have a unified approach to quantifying and partitioning taxonomic, phylogenetic and functional diversities. Most previously-proposed diversity measures can be transformed into this framework and can be better understood through it. See Chao et al. [50] for an integrated framework.

### Hill Numbers

In the traditional species diversity, only species richness and species abundances are considered. Assume there are *S* species in an assemblage and species are indexed by *i* = 1, 2, …, *S*. Let *p_i_* denote the relative abundance of the *i*th species. Hill [41] integrated species richness and species abundances into a parametric class of diversity measures later called Hill numbers, or the effective numbers of species, defined for *q*≠1 as
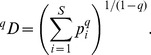
(1a)The parameter *q* determines the sensitivity of the measure to the relative abundances. When *q* = 0, ^0^ *D* is simply species richness. For *q* = 1, Eq. 1a is undefined, but its limit as *q* tends to 1 is the exponential of the familiar Shannon entropy, and is referred to as Shannon diversity in [51]:
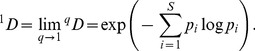
(1b)The measure ^1^ *D* weighs species in proportion to their abundances. When *q* = 2, Eq. 1a yields the inverse of the Simpson concentration which is referred to as Simpson diversity [51]:
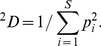
(1c)This measure places more weight on the abundant species and strongly discounts rare species. For all *q*, if *^q^D* = *u*, the diversity (of order *q*) of the actual assemblage is the same as that of an idealized assemblage with *u* equally abundant species. This is why Hill numbers are referred to as the effective numbers of species or as species equivalents.

A complete characterization of the traditional abundance-based species diversity of an assemblage with *S* species and relative abundances 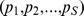 is conveyed by a diversity profile plotting *^q^D* versus *q* from *q* = 0 to *q* = 3 or 4 (beyond this it changes little) [52]. Although Hill numbers for *q*<0 can be calculated, they are dominated by the abundances of rare species and have poor statistical sampling properties. We thus restrict ourselves to the case *q*≥0 throughout the paper.

Hill [41] proved a weak version of replication principle for Hill numbers: if two equally large assemblages with no species in common have identical relative abundance distributions, then the Hill number of the pooled assemblage is doubled. Chiu et al. (Appendix B of [36]) recently proved a strong version as given in *Introduction*: the assumptions needed are that *N* assemblages with no species in common are equally large and equally diverse (relative abundance distributions may be different, unlike the weak version). Species richness is a Hill number (with *q* = 0) and obeys both versions of the replication principle, but most other traditional diversity indices do not obey even the weak version. The replication principle has been discussed for characterizing abundance-based species diversity measures [40]–[43], [53]–[56]. This replication principle will be generalized to functional diversity measures in later sections.

### Previous Distance-based Functional Diversity and Differentiation Measures

A large number of functional diversity measures have been proposed in the literature, and each measure quantifies a different aspect of species trait space. Here we mainly review those distance-based functional indices and differentiation measures that are related to our functional generalization of Hill numbers. The *FAD* measure is defined as [17]

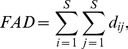
(2a)where *d_ij_* is the functional distance between the *i*th and *j*th species, *d_ij_* = *d_ji_*≥0. However, this measure does not take into account the abundances of the species, which may play an important role in the functioning of ecosystems; see [19], [57]–[63].

Functional diversity measures combining both functional distance and species abundance have been proposed [20], [32], [33], [34], [64], [65]. Rao's quadratic entropy for an assemblage with species relative abundances 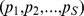 is the most widely used measure [32]:
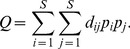
(2b)The measure *Q* is interpreted as the mean distance between any two randomly sampled individuals in the assemblage. It can also be interpreted as the abundance-weighted mean distance between two species. Here the weighting factor for a distance *d_ij_* is the product of the relative abundances, *p_i_p_j_*. This measure is an extension of the Gini-Simpson index. Although this measure has wide applications in many disciplines, *Q*, like the Gini-Simpson index, is not linear with respect to the addition of new species and thus does not obey the replication principle, causing counterintuitive results in ecological applications [35], [36], [66]. For an additive decomposition, another problem arises when the species functional distance matrix does not result in the concavity of *Q* [5], [67] (e.g. for the Gower distance matrix, in general). Then *Q* in the pooled assemblage (gamma quadratic entropy) may be smaller than the *Q* of the average of local assemblages (alpha quadratic entropy), implying *Q* in this situation could not to be used for additive decomposition [67], [68], [69]. When additive decomposition is feasible, the associated differentiation measure that has been used in the literature is the quadratic entropy excess normalized by the gamma quadratic entropy [70], [71]:
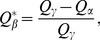
(2c)where 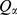 and 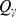 denote respectively the alpha and gamma quadratic entropy. However, when alpha quadratic entropy is high, the differentiation measure 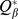 always tends to zero (implying no differentiation) regardless of distance matrices and differences in species abundances across assemblages [36]. This behavior leads to severe interpretational problems.

To fix the problems with *Q*, Ricotta and Szeidl [35] and de Bello et al. [39] made an advance by transforming *Q* to the “species equivalents”, which is the effective number of equally distinct species with a constant distance *d*_max_ for all different-species pairs; here *d*_max_ denotes the maximum value in the distance matrix. Their transformation is expressed as
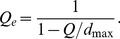
(2d)We refer to this number as “*the effective number of (equally distinct) species with maximum distance*”. Equivalently, they scale all distances so they are between 0 and 1, by dividing each distance by its maximum value in the distance matrix prior to all analyses. de Bello et al. [39], Villéger et al. [72] and Escalas et al. [73] applied the above formula to gamma and alpha quadratic entropies and obtained the corresponding effective number of species for gamma (denoted here by 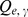) and alpha (denoted by 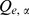), where the subscript “*e*” denotes “effective”. The resulting beta based on a multiplicative decomposition is 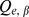 = 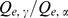. de Bello [39] further transformed this beta diversity into a normalized differentiation measure so that the resulting measure is in the unit interval [0, 1]:
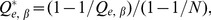
(2e)where *N* denotes the number of assemblages. Villéger et al. [72] also proposed a normalized differentiation measure:
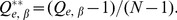
(2f)However, as we will show by examples, Eq. 2d and the two associated differentiation measures (given in Eqs. 2e and 2f) might yield un-interpretable results when they are applied to non-ultrametric distance matrices. This motivates our new approach which is valid for both ultrametric and non-ultrametric matrices.

Guiasu and Guiasu [38], [74] proposed a class of distance-weighted Gini-Simpson index as follows:
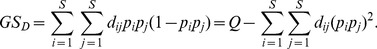
(2g)They also proposed the corresponding measure for a multiplicative decomposition. We will show that the three measures (*FAD*, *Q* and *GS_D_*) are closely related to our proposed measures. Leinster and Cobbold [75] derived a parametric class of measures sensitive to species similarity. Scheiner [63] also proposed a metric that integrates abundance, phylogeny, and function. Since both approaches are also based on a framework of Hill numbers, it is important to distinguish these two previous approaches from ours; see *Conclusion and Discussion* for more details. Neither Leinster and Cobbold's approach nor Scheiner's metric have been developed to construct normalized similarity (or differentiation) measures that can be applied to analyze datasets such as those discussed in *Examples and Applications*.

### Proposed Functional Diversity Measures

#### A Simple Framework for Ordinary Hill Numbers

We first present a simple conceptual framework for ordinary Hill numbers. Then we extend it to obtain our proposed functional Hill numbers. The intuitive interpretation of the “effective number of species” implies that if an assemblage with *S* species and species abundance vector 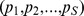 has diversity *D*, then the diversity of this actual assemblage is the same as that of an idealized reference assemblage with *D* species and species abundance (1/*D*, 1/*D*, …, 1/*D*).

Now we construct the *q*-th power sum (*q*≠1) of the abundances 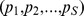 with unity weight for each species, i.e., 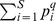; see Table 1. Taking the same function for the idealized reference assemblage, i.e., replacing *S* and 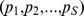 by *D* and (1/*D*, 1/*D*, …, 1/*D*) respectively, we obtain 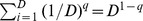. Equating the two sums shows that *D* is the Hill number of order *q*:
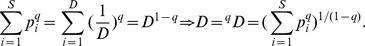
This provides a simple and intuitive derivation of Hill numbers. This derivation facilitates the extension of Hill numbers to incorporate functional distances.

**Table 1:** A framework for Hill numbers, functional Hill numbers, mean functional diversity and (total) functional diversity of a single assemblage.

	Abundance vector/matrix	weights	*q*-th power sum (*q*≠1)	Equating the two *q*-th power sums
**(1) Hill numbers**				
Actual assemblage	*S* species with relative abundance vector:	Unity weight for each species	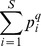	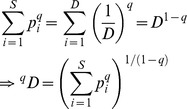
	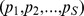	(1, 1, …., 1)		
Idealized reference assemblage	*D* equally-abundant species	Unity weight for each species	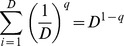	(Hill number of order *q*)
	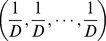	(1, 1, …., 1)		
**(2) Functional Hill number, mean functional diversity and (total) functional diversity**				
Actual assemblage	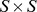 matrix of the product of relative abundances for pairs of species 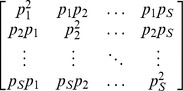	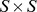 distance matrix as weight 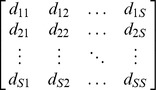	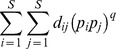	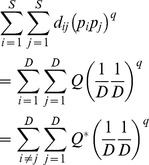
Idealized reference assemblage	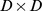 matrix of the product of equal relative abundances for pairs of species	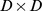 idealized distance matrix as weights	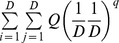 Or 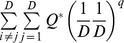	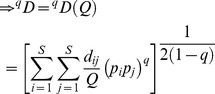
	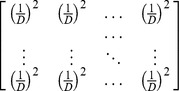	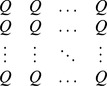		(Functional Hill number = number of rows or columns in the idealized distance matrix) 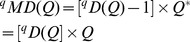
		or		
		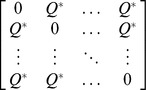		(Mean functional diversity = column/row sum in the idealized distance matrix)
		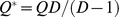		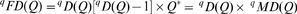
				(Total functional diversity = grand sum of the idealized distance matrix)

#### Functional Diversity Measures of an Assemblage

Let *d_ij_* denote the functional distance between the *i*th and *j*th species, with *d_ij_*≥0, and *d_ij_* = *d_ji_*. Denote the *S*×*S* symmetric pairwise distance matrix by 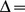
[*d_ij_*]. In our approach, species functional distance, which quantifies the proximity of species in functional trait space, can be any type of symmetric matrix. To extend Hill numbers to incorporate functional distances between species, we consider a framework based on pairs of species [38], [74]. That is, we consider a collection of all *S* ^2^ pairs of species: {(1, 1), (1, 2), (1, 3), …, (*S*, *S*)}. The joint “relative abundance” or joint probability for each species-pair (*i*, *j*) is *p_i_p_j_*. Consider the matrix 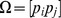, where the (*i*, *j*) element of the matrix is *p_i_p_j_* (Table 1). Note that the mean distance between any two species weighted by their joint probability is Rao's quadratic entropy defined in Eq. 2b.

Analogous to the derivation of Hill numbers, we consider the *q*-th power sum (*q*≠1) of all elements of the matrix 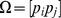 with weight *d_ij_* for species pair (*i*, *j*), i.e., 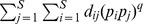. A similar concept of the “effective number of equally abundant and equally distinct species” as in ordinary Hill numbers can be applied to the functional version as follows. When species are equally distinct with a constant pairwise distance, the quadratic entropy *Q* must be equal to this constant. An assemblage with the effective number of species 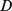 means that this assemblage has the same diversity as an idealized reference assemblage having 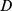 equally common and equally distinct species with a constant distance *Q* for all *S* ^2^ pairs of species. Here we have *S* ^2^ pairs because same-species pairs are included so that intraspecific variability can be considered when trait values are available at the individual level [25], [76]. (If there is no intraspecific variability, then the distance for a same-species pair is set to be 0 and a common distance 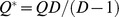 is set for different-species pairs; see Table 1. All measures derived in the following are still valid when intraspecific distance is zero, and all interpretations can be adapted to the case when there is no intraspecific variability.) For simplicity, our derivation and interpretations are mainly based on *S* ^2^ pairs of species.

Taking the same *q*-th power sum function (*q*≠1) for the idealized reference assemblage with a constant weight *Q* for all 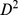 species pairs, we obtain 
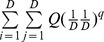
. Equating the two sums from the actual and the idealized reference assemblages leads to
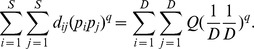
Then we can solve 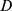 and the solution given below is denoted by 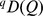:
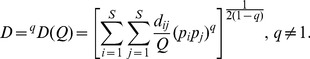
(3)For *q* = 1, we define the following limit as our measure:
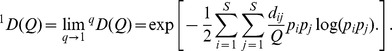
The measure 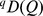 is a function of the distance matrix 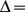
[*d_ij_*] and the joint probability matrix 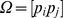. Here we express it as a function of the quadratic entropy *Q* to emphasize the important role of *Q* in the construction of other measures (see Eqs. 4a and 4b) and in the proof of the replication principle (discussed later). The measure 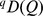 is the dimension (the number of columns or rows) of the distance matrix of the idealized reference assemblage in Table 1. We refer to it as the *functional Hill number* of order *q*. The measure 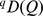 can be interpreted as “*the effective number of equally abundant and (functionally) equally distinct species*” with a constant distance *Q* for all species pairs. Thus if 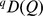 = *v*, then the functional Hill number of order *q* of the actual assemblage is the same as that of an idealized assemblage having *v* equally abundant and equally distinct species with a constant distance *Q* for all species pairs; see Table 1 for illustration.

To derive measures in units of “distance”, note that in the idealized reference assemblage, all columns and all rows have identical sums. We define the column (or row) sum as our proposed measure of *mean functional diversity (per species)*, *^q^MD*(*Q*), of order *q*:
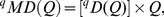
(4a)which quantifies the effective sum of pairwise distances between a fixed species and all other species (plus intraspecific distance if exists). In other words, *^q^MD*(*Q*) measures the dispersion per species in the functional trait space [18]. The product of the functional Hill numbers and the mean functional diversity thus quantifies the *total functional diversity* (or simply *functional diversity*), *^q^FD*(*Q*), in the assemblage:
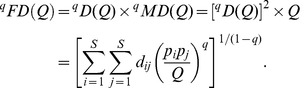
(4b)This functional diversity quantifies the effective total distance between species of the assemblage. If *^q^FD*(*Q*) = *u*, then the effective total distance between species of the actual assemblage with quadratic entropy *Q* is the same as that of an idealized assemblage having (*u*/*Q*)^1/2^ equally abundant and equally distinct species with a constant distance *Q* for all species pairs.

Consider the following special cases to intuitively understand the meaning of our functional diversity measures and their relationships with *FAD* (Eq. 2a) or *GS_D_* (Eq. 2g):

- When all species in the assemblage are equally distinct (i.e., 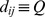 for all species pairs (*i*, *j*), for *i*, *j* = 1, 2, …, *S*), the functional Hill number 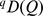 reduces to ordinary Hill number.
- For *q* = 0, 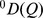 = (*FAD*/*Q*)^1/2^, ^0^ *MD*(*Q*) = (*FAD*×*Q*)^1/2^, and ^0^ *FD*(*Q*) = *FAD*, where *FAD* is defined in Eq. 2a. Thus, our measures have a direct link to *FAD*.
- If all species are equally abundant, then for any distance matrix (*d_ij_*), we have 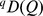 = *S*, and *^q^FD*(*Q*) = *FAD* for all orders of *q*. Therefore, when species abundances are not considered (*q* = 0) or species are equally abundant, our total functional diversity reduces to *FAD*. In the equally abundant case, we have *^q^MD*(*Q*)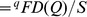, implying that our mean functional diversity is conceptually similar to the Modified Functional Attribute Diversity (MFAD) proposed by Schmera et al. [18].
- When *q* = 2, we have the following link to the weighted Gini-Simpson index 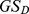 defined in Eq. 2g [38], [74]:
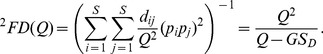
(4c)

As with the diversity profile for Hill numbers, a profile which plots 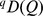, *^q^MD*(*Q*) or *^q^FD*(*Q*) with respect to the order *q* completely characterizes the information each measure gives for an assemblage. As proved in Appendix S1, all three measures 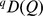, *^q^MD*(*Q*) and *^q^FD*(*Q*) are Schur-concave with respect to the product of relative abundances, implying these measures satisfy a functional version of “weak monotonicity” [45], [77], [78]. That is, if a rarest new species is added to an assemblage, then the measure *^q^FD*(*Q*) does not decrease regardless of distance matrices. Also, if a rarest new species is added to an assemblage such that the quadratic entropy remains unchanged, then all three measures do not decrease.

#### Functional Diversity Measures for a Pair of Assemblages

We next extend Rao's quadratic entropy, *FAD*, functional Hill number, mean functional diversity and total functional diversity to a pair of assemblages (say, I and II). Assume that there are *S*_1_ species in Assemblage I and *S*_2_ species in Assemblage II. Let the two sets of species relative abundances be denoted by 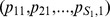 and 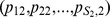 for Assemblage I and II respectively.

We first extend Rao's quadratic entropy to a pair of assemblages. Assume that an individual is randomly selected from each of the assemblages. Then the probability that the individual from Assemblage I belongs to species *i* and the individual from Assemblage II belongs to species *j* is *p_i_*_1_ *p_j_*_2_, *i* = 1, 2, …, *S*_1_, *j* = 1, 2, …, *S*_2_. The mean distance between these two randomly selected individuals is
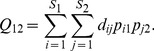
(5a)This measure can also be interpreted as the abundance-weighted mean distance between a species from Assemblage I and a species from Assemblage II, and the weighting factor is the product of their relative abundances. For simplicity, we refer to *Q*_12_ as the *mean distance between species of Assemblage I and Assemblage II*. Clearly, we have *Q*_12_ = *Q*_21_. The traditional Rao's quadratic entropy for Assemblage I is simply *Q*_11_ for the same-assemblage pair (I, I) and the quadratic entropy for Assemblage II is simply *Q*_22_ for the same-assemblage pair (II, II).

We can apply a similar approach to that in Table 1 by conceptually thinking that there are two idealized assemblages, and each assemblage includes 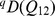 equally abundant and equally distinct species such that the two actual assemblages and the two idealized assemblages have the same value of a given diversity measure. Replacing the joint probability matrix 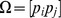 in Table 1 with the *S*_1_×*S*_2_ matrix 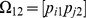 and using parallel derivations, we obtain the following functional Hill number for Assemblage I and Assemblage II:
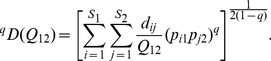
(5b)This measure is interpreted as “the effective numbers of equally abundant and equally distinct species in each of two assemblages, with a constant distance *Q*_12_ between species of Assemblage I and Assemblage II”. We also define the mean functional diversity of Assemblages I and II as 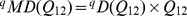, which quantifies the effective sum of pairwise distances between a fixed species in one assemblage and all species in the other assemblage. Then the product of 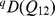 and 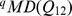 quantifies the *total functional diversity* (or simply *functional diversity*) *of* Assemblage I and Assemblage II as
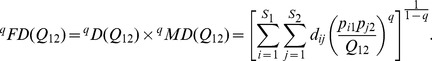
(5c)In the special case of *q* = 0, the above total functional diversity reduces to the total sum of all pairwise distances between species of Assemblage I and Assemblage II. Since *Q*_12_ is not involved in the measure for *q* = 0, we denote ^0^ *FD*(*Q*_12_)≡*FAD*_12_, which represents an extension of Walker's *FAD* to a pair of assemblages. Thus *FAD*_11_ is identical to *FAD* for Assemblage I and *FAD*_22_ is identical to *FAD* for Assemblage II. Also, we have the following relationship:
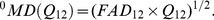
(5d)


#### Replication Principle

We generalize the concept of the replication principle to a functional version and show that the proposed functional Hill numbers and the mean functional diversity both satisfy the replication principle. Consequently, the product of these two measures (i.e., our proposed total functional diversity) satisfies a quadratic replication principle (i.e., the total functional diversity of the pooled assemblage is *N* ^2^ times that of any individual assemblage.) A general proof of the replication principle for *N* completely distinct assemblages is given in Appendix S1. Throughout this paper, *N* assemblages are *completely distinct* if there are no shared species (and thus no shared species pairwise distances).

To simplify the concept, here we present the replication principle only for two assemblages. Assume that two equally large and completely distinct assemblages are pooled. Let *Q*_11_, *Q*_12_, *Q*_21_, and *Q*_22_ denote respectively the mean distance between species of the four pairs of assemblages, (I, I), (I, II), (II, I) and (II, II). Assume that the functional Hill number of order *q* for all of the four pairs of assemblages is a constant 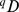. When the two assemblages are combined, the quadratic entropy in the pooled assemblage becomes 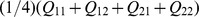 and the functional Hill number of order *q* in the pooled assemblage is doubled. Consequently, if we further assume that the four mean distances (*Q*_11_, *Q*_12_, *Q*_21_ and *Q*_22_) are identical, then the mean functional diversity in the pooled assemblage is also doubled, and the total functional diversity is quadrupled; see Appendix S1 for a general proof for *N* assemblage.

In Guiasu and Guiasu's work on the quadrupling property [74], they proved a weak version of the quadrupling property for their proposed weighted Gini-Simpson type index (Eq. 4c) when two equally large and completely distinct assemblages (I and II) are pooled. They assume that the joint probability matrices for the four pairs of assemblages, (I, I), (I, II), (II, I) and (II, II), are identical, and also assume that the species distance matrices for the four pairs of assemblages are also identical. The latter assumption implies the *FAD* for the four pairs is a constant (say, *A*), i.e., *FAD*_11_ = *FAD*_12_ = *FAD*_21_ = *FAD*_22_≡*A*. This weak version can be directly used to understand why the functional diversity of order zero (i.e., *FAD*) satisfies a quadrupling property. In this simple case, consider the distance matrix of the pooled assemblage when the two actual assemblages have no species shared. It is readily seen that the total distance between species in the pooled assemblage is quadrupled because the *FAD* in the pooled assemblage is *FAD*_11_+*FAD*_12_+*FAD*_21_+*FAD*_22_ = 4×*A*. As shown in the proof (Appendix S1), our replication principle is a strong version in the sense that there are no restrictions on the joint probability matrices and on the distance matrices.

### Partitioning Functional Diversity Measures

Assume that there are *N* assemblages defined by the investigator. The functional Hill number 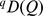 (Eq. 3), mean functional diversity *^q^MD*(*Q*) (Eq. 4a) and total functional diversity *^q^FD*(*Q*) (Eq. 4b) of the pooled assemblage can each be decomposed into independent alpha and beta components. Here we adopt the decomposition method developed by Chiu et al. [36]. We first briefly review Chiu et al.'s method for partitioning Hill numbers in order to provide necessary backgrounds. To calculate the gamma Hill numbers, species abundances are pooled over assemblages; the gamma diversity is Hill numbers computed from the species relative abundances in the pooled assemblage. However, as noted by de Bello et al. [39], how to define species relative abundances in the pooled assemblage depends on how we pool data over assemblages. The pooling scheme depends on the objective of the study. If the objective is to compare absolute abundances among assemblages (given the total abundance in the pooled assemblage), we should pool individuals over assemblages; if the objective is to compare relative abundances among assemblages, we should pool relative abundances of individual assemblages. These two kinds of pooling schemes lead to different relative abundances in the pooled assemblage. (The former is equivalent to an assemblage-size-weighted pooling scheme for relative abundances, whereas the latter naturally reduces to the equal-assemblage-weighted pooling scheme.) See Chao et al. [50, their Appendix 2] for a simple example for illustration.

Suppose in the pooled assemblage there are *S* species indexed by 1, 2, …, *S*. To incorporate both kinds of pooling schemes into our framework, we define *z_ik_* as any measure of species “abundance” of the *i*th species in the *k*th assemblage, *i* = 1, 2, …, *S*, *k* = 1, 2, …, *N*. Some of the *z_ik_* may be zero. The measure *z_ik_* can be absolute abundances, relative abundances, incidence, biomasses, cover areas or any other species importance measure. Define 
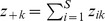
 as the size of the *k*th assemblage. Let 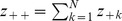 be the total abundance in pooled assemblage and 
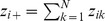
 be the total abundances of the *i*th species in the pooled assemblage. Then the species relative abundance set in the pooled assemblage for both pooling schemes can be expressed as {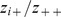; *i* = 1, 2,…, *S*}. Note that if we pool relative abundances over assemblages instead of absolute abundance, we have the special case that *z*_+*k*_ = 1 and *z*_++_ = *N*.

The abundance-based gamma diversity is Hill numbers computed from the species relative abundances {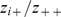; *i* = 1, 2,…, *S*} and is interpreted as the effective number of species in the pooled assemblage. The traditional definition of alpha diversity is “the mean of the diversities of individual assemblages”. Routledge [55] and Jost [43] each derived a mathematical formula for alpha diversity based on this traditional definition and obtained the corresponding multiplicative beta component. As Chiu et al. [36] indicated, this traditional approach to alpha diversity based on Hill numbers leads to a beta that can only be used to produce differentiation measures to compare species *relative* abundances, but not *absolute* abundances. This is because in the framework of Hill numbers, diversity is a function of relative abundances only, and thus “the mean of individual diversities” lose information about absolute abundances. Chiu et al. [36] expanded the conventional concept of alpha and proposed a modified definition for abundance-based alpha diversity: “alpha diversity is the effective number of species per assemblage” so that the resulting beta can be transformed to quantify the differentiation (or similarity) among *N* sets of vectors 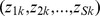, *k* = 1, 2, …, *N*, for any measure of species importance *z_ik_*, including absolute abundances. Based on this expanded definition, Chiu et al. derived a new formula for abundance-based alpha diversity.

When the data represent species relative abundances (i.e., equal-weight for assemblages), all three alpha formulas (Routledge, Jost and Chiu et al.) are identical. They differ, however, when the data represent species absolute abundances (i.e., assemblage-size as weight); Routledge's beta may exceed *N* and Jost's beta may be less than unity (i.e., gamma may be less than alpha) if *q*≠1. Also, for *q* = 0, Routledge's alpha and beta are not independent [49]. Chiu et al.'s new formula of the abundance-based alpha diversity has the following advantages: (1) it leads to a beta that can be applied to compare *any* types of data (*z_ik_*), depending on the investigator's objective; (2) gamma is always greater than or equal to alpha for *all* orders *q*≥0; (3) beta is always between unity (when all assemblages are identical in species absolute abundances) and *N* (when the *N* assemblages have no species in common); and (4) alpha and beta components are independent for *all* orders *q*≥0.

Based on species abundance (*z_ik_*) and a species pairwise distance matrix, we now extend Chiu et al. approach to decompose the functional diversity *^q^FD*(*Q*) of the pooled assemblage into within-assemblage component (functional alpha diversity) and between-assemblage component (functional beta diversity). As with the partition of Hill numbers, the functional gamma diversity of order *q* is based on the distance matrix [*d_ij_*] and the relative abundance 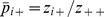 in the pooled assemblage. It then follows from Eq. 4b, the *functional gamma diversity* of order *q* can be written as
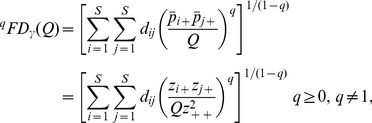
(6a)where 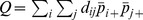 is the quadratic entropy in the pooled assemblage. The limit when *q* approaches unity exists and is equal to
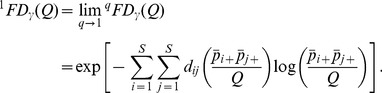
(6b)The functional gamma diversity is interpreted as the effective total distance between species in the pooled assemblage with a constant distance *Q* for all species pairs.

We follow Chiu et al.'s definition of alpha diversity to define the functional alpha diversity as the effective total distance between species of a pair of individual assemblages. Then we obtain (details of derivation are provided in Appendix S2) the *functional alpha diversity* of order *q*:
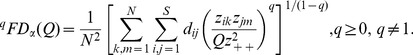
(7a)

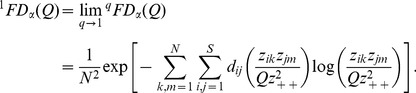
(7b)Note that in our alpha formula, *Q* still refers to the quadratic entropy for the *pooled* assemblage. When relative abundances are the relevant quantities for the investigative question, we simply define the measure *z_ik_* as the *i*th species relative abundance in the *k*th assemblage. Then 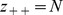 and thus *z*_++_ is replaced by *N* in all of the above formulas, Eqs. 6a, 6b, 7a and 7b.

As with ordinary Hill numbers [36], [43], the complete partitioning of functional gamma diversity into independent within- and between-assemblage (alpha and beta) components is multiplicative. That is, the *functional beta diversity* is the ratio of functional gamma to functional alpha diversities:
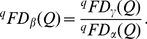
(8)The decomposition procedures for the other two measures are generally parallel and interpretations are similar. A summary of the decomposition of the three measures with interpretations is given in Table 2 and the formulas for decomposing functional Hill numbers are provided in Appendix S2. Note that for functional Hill number and mean functional diversity, their beta components are identical, i.e., 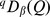 = 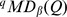. Also, we have 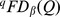 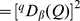.

**Table 2:** Decomposition of the functional Hill number 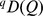 (Eq. 3), the mean functional diversity *^q^MD*(*Q*) (Eq. 4a) and the (total) functional diversity *^q^FD*(*Q*) (Eq. 4b) along with interpretations.

Measure	Functional Hill number 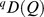	Mean functional diversity 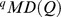	(Total) functional diversity 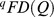
Gamma	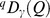	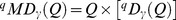	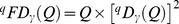
	The effective number of species in the pooled assemblage	The effective mean distance between species in the pooled assemblage	The effective total distance between species in the pooled assemblage (Eq. 6)
Alpha	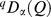	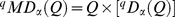	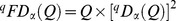
	The effective number of species in an individual assemblage	The effective mean distance between species in an individual assemblage	The effective total distance between species in a pair of local assemblage (Eq. 7)
Beta	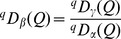	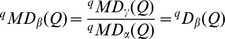	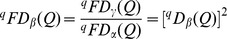
	The effective number of equally large and completely distinct assemblages	The effective number of equally large and completely distinct assemblages	The effective number of equally large and completely distinct assemblage pairs

For each of the three measures (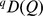, *^q^MD*(*Q*) and *^q^FD*(*Q*)), the gamma value is always greater than or equal to the corresponding alpha component for all orders *q*≥0 and all distance matrices; see Appendix S2 for a proof. When *N* assemblages are identical in species identities and abundance, the beta components of all three measures take their minimum value of unity. When all assemblages are completely distinct (no shared species and thus no shared pairwise distances), we have 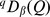 = 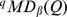 and both attain the maximum value of *N*, and 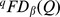 attains the maximum value of *N* ^2^. The functional beta Hill number, 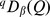, thus quantifies the effective number of *equally large and completely distinct assemblages*. The functional beta diversity, 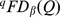, quantifies the effective number of *equally large and completely distinct pairs of assemblages*. In Appendix S2, we show that 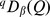 is always between unity and *N*; and 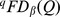 is always between unity and *N* ^2^. Thus, the range of each beta component is independent of the corresponding alpha component, implying that the alpha and beta components based on the multiplicative partitioning for each of the three functional diversity measures (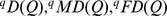) are unrelated (or independent).

We also note the following properties:

1. When all species are equally distinct, the functional beta Hill numbers 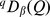 reduce to the beta diversity for ordinary Hill numbers, and the functional beta diversity 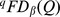 reduces to the squared beta diversity of ordinary Hill numbers [36].
2. When *q* = 0, we have 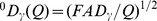, 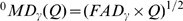 and 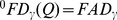, where 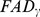 denotes the sum of pairwise distances in the pooled assemblage. For the alpha components, we have 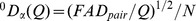, 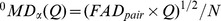 and 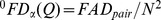, where *FAD_pair_* is the sum of *FAD*s over all possible pairs of assemblages (there are *N* ^2^ pairs of assemblages). So the functional alpha diversity for *q* = 0 is the average of *FAD* per pair of assemblages. Therefore, we have 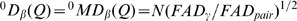 and 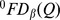 = 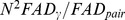.
3. For the special case *q* = 1 and *q* = 2, we will present formulas for some special transformations of the functional beta diversity in the next section.

### Four Classes of Normalized Functional Similarity Measures

Our functional beta components, 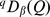
( = 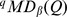) and 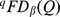, all quantify pure functional differentiation among the *N* assemblages and their ranges depends only on *N*. Thus, each can be transformed to obtain the normalized similarity and differentiation measures in [0, 1] so that the dependence on *N* can be removed [36], [43], [49], [79]. As stated in the preceding section and proved in Appendix S2, the range of 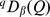 is different from that of 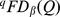, so the transformations to normalized similarity measures in [0, 1] are thus different for these two beta components. The similarity measures based on 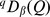 quantify *species-overlap* from different perspectives, whereas the similarity measures based on 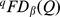 quantify *distance-overlap* from different perspectives. In most applications of functional diversity, we suggest using the distance-overlap similarity measures and their corresponding differentiation measures. We now describe the two major classes of normalized distance-overlap measures based on 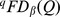; see Table 3 for all formulas and Appendix S3 for details.

**Table 3:** Two major classes of distance-overlap (or similarity) measures and their special cases based on the functional beta diversity 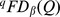.

Order *q*	Local distance-overlap	Regional distance-overlap
	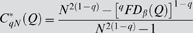	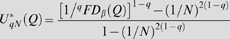
*q* = 0	Func-Sørensen	Func-Jaccard
	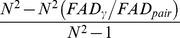	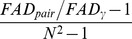
*q* = 1	Func-Horn	
	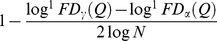	
*q* = 2	Func-Morisita-Horn	Func-regional-overlap
	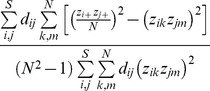	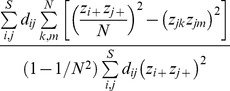

The corresponding differentiation measures are the one-complements of the similarity measures. (The indices *i* and *j* are used to identify species, *i*, *j* = 1, 2, …, *S*, and the indices *k* and *m* are used to identify assemblages, *k*, *m* = 1, 2, …, *N*.)

Notation.

*z_ik_* = the abundance of the *i*th species in the *k*th assemblage, 
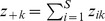
, 
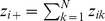
, and 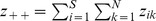; see text for details. 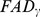 = sum of the pairwise distances between species in the pooled assemblage; 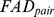 = sum of *FAD* over all possible pairs of assemblages (there are *N* ^2^ pairs of assemblages). *S* = species richness in the pooled assemblage. 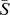 = average species richness per assemblage.

(1) A class of local distance-overlap measures from the perspective of a pair of local assemblages
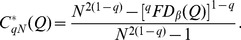
(9a)Here “local” refers to a property of a pair of individual assemblage. This measure gives the effective average proportion of the species pairwise distances in a pair of local assemblages that are shared with all other pairs of assemblages. See Appendix S3 for the interpretation of the “effective” average proportion.

We first give the special case of *q* = 0 to intuitively explain its properties: since 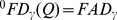 and 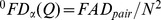, the measure 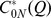 reduces to
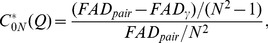
(9b)where 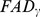 and *FAD_pair_* are defined in the preceding section. In this expression, the denominator is the zero-order functional alpha diversity, which is the average of *FAD*s over all *N* ^2^ assemblage pairs; the numerator is the average of all repeated pairwise distances in the *N* ^2^−1 pairs of assemblages (excluding the assemblage-pair in which a pairwise distance is first counted). The measure 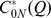 thus quantifies the proportion of repeated distances in a pair of local assemblages. This interpretation is conceptually similar to the traditional Sørensen similarity index. The difference is that here we consider “assemblage-pairs” in functional distance-overlap measures rather than “individual assemblage” as in the traditional measure. Thus, this index can be regarded as an extension of the Sørensen index to functional similarity. Therefore, the measure 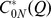 is referred to as “func-Sørensen” in Table 3. For *q* = 1, this local distance-overlap measure is called “func-Horn” in Table 3 because when all distances are identical and (*z_ik_*) represents species relative abundance within each assemblage, it reduces to the classical Horn measure [80]. For *q* = 2, this measure is called “func-Morisita-Horn” in Table 3 because its interpretation is generally similar to the classic Morisita-Horn measure [81]. See Appendix S3 for more details.

(2) A class of regional distance-overlap measures in the pooled assemblage
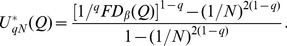
(10a)Here “regional” refers to a property of the pooled assemblage. This class of measures differs from the local distance-overlap measures by taking a regional perspective. It quantifies the effective proportion of the species pairwise distances in the pooled assemblage that are shared with all pairs of local assemblages (Appendix S3).

For the special case of *q* = 0, the measure 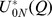 reduces to
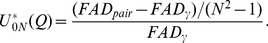
(10b)Again, the interpretation is similar to the measure 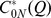 (in Eq. 9b) except that the denominator is replaced by the gamma *FAD*. Thus, our index can be regarded as an extension of the Jaccard index to functional similarity. This is why the measure 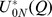 is referred to as the *N*-assemblage “func-Jaccard” in Table 3. Since 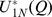 = 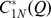, the measure 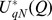 for *q* = 1 is also called “func-Horn” measure. For *q* = 2, this measure is referred to as “func-regional-overlap” measure; see Appendix S3.

As shown in Chiu et al. [36], we can also define two additional classes of functional distance-overlap measures. First, a class of functional distance-homogeneity measures which is a linear function of the inverse of the functional beta diversity:
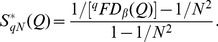
(11)Second, we have a class of measures which is a linear function of the functional beta diversity:
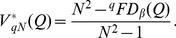
(12)Its complement quantifies the functional distance-turnover rate.

All the four classes of similarity measures are continuous in *q*≥0, so a functional similarity or differentiation profile as a function of *q* can be made for any of them. We suggest using this method for conveying complete information about the functional similarity or differentiation of a set of assemblages. It is thus sufficient to focus on the two major classes (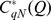 and 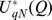) because they include 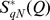 and 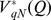 as special cases for *q* = 0 and 2. See Example 3 for illustrative profiles.

Our decomposition presented above is based on the multiplicative scheme. We can also apply the additive decomposition to each of the three measures, 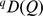 (Eq. 3), *^q^MD*(*Q*) (Eq. 4a) and 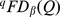 (Eq. 4b). For example, we can define the “functional diversity excess” as 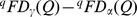. The excess quantifies the effective total distances between species in the pooled assemblage not contained in a typical pair of local assemblages. As with ordinary Hill numbers [49], the functional diversity excess depends not only on the number of assemblages *N*, but also on the functional alpha diversity. Consequently, the excess measure cannot be directly applied to compare the similarity or differentiation among assemblages across multiple sets of assemblages even if the numbers of assemblages in these multiple regions are the same. We can eliminate these dependences by using appropriate normalizations [49]. In Appendix S4, we show that after proper normalizations, the multiplicative approach and additive approach both lead to the same four classes of normalized functional similarity and differentiation measures presented above. Thus, a consensus can be achieved on functional similarity and differentiation measures, including those measures given in Table 3.

### Examples and Applications

To examine the performance of our functional diversity measures and to compare our proposed similarity and differentiation measures with previous indices, we use both artificial distance matrices (Examples 1 and 2) and real data (Example 3) for illustration. Although the distance matrices considered in our artificial examples are simple, they provide transparent answers so that we can clearly examine the performance of measures. Any meaningful differentiation measure should work properly for *all* matrices. If a functional diversity or similarity/differentiation measure cannot yield logical and sensible results for simple matrices, we would not expect it to work for complicated cases. The more complicated distance matrix calculated from real species traits is used in Example 3 for illustration.

In our comparisons, we consider various functional differentiation measures: (1) a differentiation measure (Eq. 2c) based on the traditional additive decomposition of quadratic entropy; (2) two differentiation measures (Eqs. 2e and 2f) based on the effective number of species with maximum distance; and (3) the proposed distance-based differentiation measures derived from our functional beta diversity (in Table 3 of this paper). Appendix S5 presents a simple example to show that the traditional measure based on the additive partitioning of the quadratic entropy (Eq. 2c) cannot work properly even for a very simple functional distance matrix; see Chiu et al. [36] for theoretic discussions and more examples.

#### Example 1: Effect of Functional Distances on Differentiation Measures

Consider two assemblages (I and II). Each assemblage contains 20 species, with 12 shared species and 8 non-shared species. There are 28 species in the pooled assemblage. For each assemblage, we first consider the equally abundant case in order to examine how differentiation measures vary with functional distances. (Two non-equally-abundant cases are given in Appendix S5.) The classical Sørensen-type dissimilarity index (the proportion of non-shared species in an individual assemblage) is 8/20 = 0.4. (The abundance-based local differentiation measure based on Hill numbers is 0.4 for all *q*≥0; see [36].) The classical Jaccard-type dissimilarity index (the proportion of non-shared species in the pooled assemblage) is 1–12/28 = 0.571; see Table 4 for abundance-based regional differentiation measure based on Hill numbers [36]. For functional differentiation measures, the quantifying target is shifted to the proportion of the total non-shared distances (incorporating abundances if *q*>0) in an individual assemblage (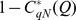) or in the pooled assemblage (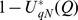).

**Table 4:** Comparison of various differentiation measures for Matrix I (with 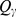 = 0.48, 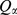 = 0.47) and Matrix II (with 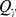 = 0.167, 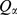 = 0.102) based on abundance and function (*A&F*), on function (*F*) only, and abundance (*A*) only.

Measure	Order	Matrix I			Matrix II		
		*A&F*	*F*	*A* #	*A&F*	*F*	*A* #
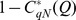	*q* = 0	0.324	0.324	0.4	0.579	0.579	0.4
	*q* = 1	0.408	---	0.4	0.628	---	0.4
	*q* = 2	0.491	---	0.4	0.678	---	0.4
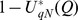	*q* = 0	0.657	0.657	0.571	0.846	0.846	0.571
	*q* = 1	0.408	---	0.4	0.628	---	0.4
	*q* = 2	0.194	---	0.25	0.345	---	0.25
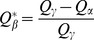	*q* = 2	0.002			0.388		
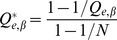	*q* = 2	0.004			0.145		
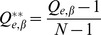	*q* = 2	0.002			0.078		

# Differentiation measures are the abundance-based local differentiation measure (1−*C_qN_*) and regional differentiation measure (1−*U_qN_*) obtained from partitioning Hill numbers [36];

--- No measures for *q* = 1 and *q* = 2 because species abundances are not considered for measures based on function (*F*) only.

We generated two contrasting types of distance matrices (Matrix I and Matrix II). Both matrices are displayed in Appendix S6. For easy presentation, species are indexed by 1, 2, …, 28 in the pooled assemblage. Assemblage I includes Species 1–20, and Assemblage II includes Species 9–28 (Species 9–20 are shared). In Matrix I, the distances for two species within an assemblage follow the same distribution as those for species from the pooled assemblage so that the alpha quadratic entropy *Q_α_* (the average distance between any two individuals within an assemblage) is close to the gamma quadratic entropy 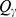 (the average distance between any two individuals in the pooled assemblage). In this case, we expect that any meaningful functional differentiation measure is largely determined by species abundances. In Matrix II, the gamma quadratic entropy 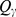 is much higher than the alpha quadratic entropy *Q_α_*, as described below. Consequently, we expect that functional distances should play an important role in characterizing functional differentiation.

- **Matrix I.** All the species pairwise distances in the 28×28 distance matrix of the pooled assemblage were generated from a beta (4, 4) distribution, which is a symmetric distribution with respect to 0.5. In this case, the alpha quadratic entropy (*Q_α_* = 0.47) is close to the gamma quadratic entropy (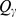 = 0.48).
- **Matrix II.** We constructed the 28×28 distance matrix by generating substantially larger distances for pairs of “non-shared species” (s1, s2), where the first species s1 is a non-shared species in Assemblage I, and the second species s2 is a non-shared species in Assemblage II. The distances for such pairs of non-shared species were generated from a uniform (0.8, 1) distribution whereas the distances for other species pairs were generated from a uniform (0, 0.2) distribution. We have *Q_α_* = 0.102 and 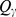 = 0.167. There is large relative difference between *Q_α_* and 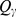, as reflected by the high relative difference (with respect to the alpha) of 63.7%.

In Table 4, we first compare separately for Matrix I and Matrix II the differentiation measures incorporating both abundance and function (*A*&*F*), function (*F*) only, and abundance (*A*) only. The measures considering both (*A*&*F*) are based on our proposed measures 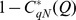 and 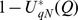 (with formulas in Table 3) derived from the functional beta diversity. The measure based only on function only (*F*) does not consider abundance, so it is identical to the zero-order of the measure considering *A*&*F*. The measures considering abundance only (*A*) refer to the abundance-based local differentiation measure (1−*C_qN_*) and regional differentiation measure (1−*U_qN_*) based on partitioning Hill numbers ([36], p. 31).

Comparing the column under *A*&* F* and the column under *A* within Matrix I, we find for each fixed order of *q* = 0 and *q* = 2 that there is appreciable difference between these two values (*A*&* F* and *A*) but the difference is limited to some extent (relatively to the corresponding difference for Matrix II); the difference is very little for *q* = 1. This is valid for both differentiation measures 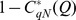 and 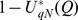. Thus, for Matrix I (with similar distributional pattern of distances for all species pairs), functional differentiation is largely determined by species abundance pattern and function plays a minor factor.

In contrast, for Matrix II, the impact of function on our differentiation measures is clearly seen for both measures 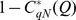 and 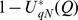 by noting that our measure considering both (*A*&*F*) is much higher than the corresponding measure considering *A* for all orders *q* = 0, 1 and 2. This is because the functional distances for pairs of non-shared species are substantially larger than those of other species pairs, leading to a large increase in the proportion of non-shared distances in an assemblage (as reflected in our local distance-differentiation measure 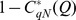), and also in the pooled assemblage (as reflected in our regional distance-differentiation measure 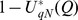). In this case, function has profound effect on characterizing functional differentiation. Since the two measures (*A*&*F* and *A*) of *q* = 1 differ little for Matrix I whereas they differ substantially for Matrix II, their difference is a potentially useful indicator for the effect of function. All the above findings not only hold for equally abundant species as the example presented here but also are generally valid if species abundances are heterogeneous; see Appendix S5 for two heterogeneous cases.

For both matrices the proposed measures exhibit moderate differentiation between the two assemblages for Matrix I and moderate to high differentiation for Matrix II. For example, our proposed measure, 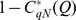, yields values 0.324 (for *q* = 0), 0.408 (for *q* = 1) and 0.491 (for *q* = 2) for Matrix I. The corresponding three values for Matrix II are 0.579 (for *q* = 0), 0.628 (for *q* = 1) and 0.678 (for *q* = 2). Table 4 reveals that the differentiation measure based on the additive partitioning of the quadratic entropy exhibits an unreasonably low differentiation value of 0.002 for Matrix I. As shown in reference [36], this measure does not properly quantify functional differentiation; also see the example in Appendix S5. The two measures based on the effective number of species with maximum distance (Eqs. 2e and 2f) for both matrices also show unreasonably low differentiation. For Matrix I, the measure in Eq. 2e gives a value of 0.004 and the measure in Eq. 2f gives a value of 0.002, implying that there is almost no differentiation among the two assemblages. These are counter-intuitive and unexpected values because function has almost no effect and thus all measures for Matrix I should yield close results to those based on abundances only (the column under *A* in Table 4). This example also helps show that the measures in Eqs. 2e and 2f cannot be applied to non-ultrametric cases, as the two matrices are both non-ultrametric (Appendix S6). Similar findings about substantially low functional differentiation are also revealed in other papers [82], [83]. For Matrix II, each of the two previously developed measures (Eqs. 2e and 2f) is also substantially lower than our proposed differentiation measure considering both (*A*&*F*). More evidence from other perspectives is provided in Example 2 below.

#### Example 2: Ultrametric vs. Non-ultrametric Distance Matrices

In this example, we compare the performance of various differentiation measures when they are applied to an ultrametric matrix (Case I in Table 5) and a non-ultrametric matrix (Case II in Table 5). Each matrix represents a distance matrix for a pooled assemblage of four species. In each case, there are two completely distinct assemblages (no species shared). There are two equally common species (*a*, *b*) in the first assemblage, and two equally common species (*c*, *d*) in the other assemblage. We use this simple example to show that the effective approach based on the effective number of species with maximum distance (Eq. 2d) and the associated differentiation measures (Eqs. 2e and 2f) may lead to un-interpretable conclusions if they are applied to non-ultrametric distance matrices.

**Table 5:** Comparison of various differentiation measures between two assemblages for an ultramteric distance matrix (Case I below) and a non-ultrametric distance matrix (Case II below).

Measure	Ultrametric distance matrix (Case I)	Non-ultrametric distance matrix (Case II)
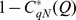	1 (for all *q*≥0)	1 (for all *q*≥0)
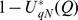	1 (for all *q*≥0)	1 (for all *q*≥0)
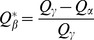	0.6	0.826
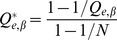	1	0.559
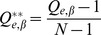	1	0.388

Assume the two assemblages are completely distinct. There are two equally common species (*a*, *b*) in the fisrt assemblage, and two equally common species (*c*, *d*) in the second assemblage. In the pooled assemblage, there are four species (*a*, *b*, *c*, *d*) with relative abundances (0.25, 0.25, 0.25, 0.25). As explained in the text, we expect that the differentiation for Case II should not be lower than that for Case I. See Appendix S5 for a non-completely-distinct case.

Case I: An ultrametric distance matrix for four species (*a*, *b*, *c*, *d*) with 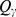 = 0.125, 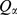 = 0.05.
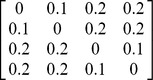

Case II: A non-ultrametric distance matrix for four species (*a*, *b*, *c*, *d*) with 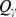 = 0.288, 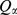 = 0.05.
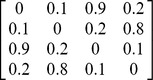

Comparing the two distance matrices, we see that the two matrices are identical except for the distances for the two pairs, (*a*, *c*) and (*b*, *d*). The distance between Species *a* and Species *c* is 0.2 in Case I but it is increased to 0.9 in Case II; the distance for Species *b* and Species *d* is 0.2 in Case I but it is increased to 0.8 in Case II. Thus, when the matrix is changed from Case I to Case II, the distance for any two species in different assemblages is either increased or kept as the same, whereas all the distances for species in the same assemblage are kept the same. By intuition and by theory for our measures (Proposition S2.2 in Appendix S2), any sensible differentiation measure should not decrease.

In Table 5, we compare various differentiation measures between the two assemblages separately for Case I and Case II. The measures based on Eqs. 2e and 2f both produce a maximum differentiation of unity for Case I. This is intuitively understandable because the two assemblages are completely distinct and all distances for two species in different assemblages are higher than the distances for two species within an assemblage. In both Case 1 and Case II, the proposed differentiation measures, 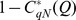 and 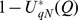, attain the maximum differentiation of unity for all orders of *q*, showing the differentiation does not decrease from Case I to Case II. However, the two differentiation measures (Eqs. 2e and 2f) for Case II give unexpectedly lower differentiation than that of Case I. This example shows why application of Eq. 2d and the associated differentiation measures (Eqs. 2e and 2f) to non-ultrametric cases might be misleading. Although the measure based on additively partitioning quadratic entropy (Eq. 2c) yields higher differentiation for Case II, we have demonstrated its counter-intuitive behavior in Appendix S5 and in Example 1.

In this example, we specifically use the extreme case that two assemblages are completely distinct (no shared species) for illustrative purpose. A more general property of monotonicity is proved in Appendix S2 (Proposition S2.2): any differentiation measure based on our functional beta diversity is a non-decreasing function with respect to the distance of any non-shared species pair regardless of species abundance distributions. This property of monotonicity implies that the differentiation measure including 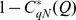 and 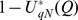 do not decrease if the distance for a non-shared species pair becomes larger even if the two assemblages are not completely distinct. In Appendix S5, we provide a supplementary example in which there are shared species between assemblages; our proposed measures yield the expected property of monotonicity, while the two previous differentiation measures (Eqs. 2e and 2f) do not.

#### Example 3: A Real Functional Distance Matrix for Dune Vegetation

We apply our proposed measures to the real data discussed by Ricotta et al. in [84]. The data contain a total of 43 vascular plant species collected from 272 random vegetation plots of 2×2 m in size during the period 2002–2009 in three successively less extreme fore dune habitats: embryo dunes (EM; 17 species in 70 plots), mobile dunes (MO; 39 species in 131 plots) and transition dunes (TR; 42 species in 71 plots) along the Tyrrhenian coast, where EM is closest to the sea, MO is between EM and TR, and TR is farthest from the sea; see [85], [86], [87] for details. There are 17 shared species (out of a total of 39 species) between EM and MO, 16 shared species (out of a total of 43 species) between EM and TR, and 38 shared species (out of a total of 43 species) between MO and TR. In each habitat, we pooled species abundance data over plots and applied various diversity and differentiation measures based on the species relative abundances (Table S5.4 in Appendix S5) in the three type habitats.

All species were described by a set of sixteen functional traits which include seven quantitative variables: plant height, leaf size, leaf thickness, seed mass, seed shape, leaf dry mass and specific leaf area, together with nine categorical variables: life form, growth form, leaf texture, dispersal mode, leaf persistence, plant life span, pollination system, clonality and flowering phenology. Based on these sixteen traits, the species distance matrix in the pooled assemblage was calculated by a Gower mixed-variables coefficient of distance with equal weights for all traits [71]. The Gower species pairwise distance matrix of the pooled assemblage is provided in Appendix S6. The matrix and the three sub-matrices (corresponding to those of three habitats) are all non-ultrametric. The two idealized examples (Example 1 and Example 2) just given showed that previously-proposed functional differential measures led to unexpected conclusions when applied to non-ultrametric matrices. This real example shows how such mathematical problems can lead to misinterpretation of important ecological patterns.

**Figure 1:**
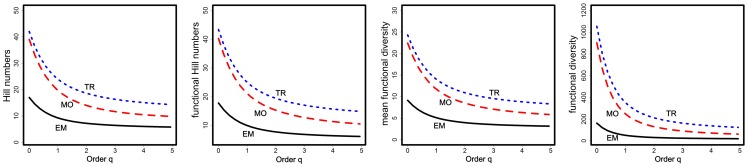
Diversity profiles as a function of order *q* for ordinary Hill numbers *^q^D* (left panel), functional Hill numbers 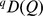 (the second panel from the left), mean functional diversity *^q^MD*(*Q*) (the third panel from the left) and (total) functional diversity *^q^FD*(*Q*) (right panel) for three habitats (TR, MO, and EM). All the profiles show a consistent diversity pattern about the ordering of the three habitats: TR>MO>EM.

For each of the three habitats, we present four diversity measures: ordinary Hill numbers *^q^D* (Eq. 1a), our functional Hill number 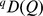 (Eq. 3), mean functional diversity *^q^MD*(*Q*) (Eq. 4a) and functional diversity *^q^FD*(*Q*) (Eq. 4b). The diversity profiles for the four diversity measures as a function of order *q* are shown in Fig. 1. A consistent pattern is revealed in Fig. 1: EM has the lowest diversity, MO is intermediate, and TR has the highest diversity. This pattern is valid for all orders of *q*, and is expected from ecologists' perspectives [84]. The EM is closest to the sea, and hence exposed to wind disturbance, flooding, salt spray, and other harsh environmental factors. Therefore, the assemblage in the EM is mainly composed of a few specialized pioneer species with similar functional traits (as reflected by the value of quadratic entropy, which is respectively 0.513, 0.556, and 0.561 in EM, MO and TR) to adapt the extreme environment, leading to the lowest functional diversity in this habitat. The vegetation of the MO is less affected by harsh environment factors, so the vegetation presents more diverse species composition, resulting in larger functional distances and thus higher functional diversity. The species richness and evenness in the TR are the highest among the three habitats and the vegetation of TR is even more weakly constrained by these environmental factors, supporting an even higher functional diversity. The diversity pattern for Hill numbers is similar to those based on functional diversity measures, as will be discussed later. In each of the three functional diversity profiles (the two middle panels and the right panel of Fig. 1), the initial value (i.e., the value for *q* = 0) represents the diversity when only function is considered.

The formula in Eq. 2d produces much lower values of species equivalents: 2.94 (EM), 3.39 (MO) and 2.95 (TR), substantially lower than the corresponding functional Hill numbers (*q* = 2): 7.72 (EM), 15.27 (MO), 19.42 (TR); see the second panel of Fig. 1. Moreover, the number of species equivalents from Eq. 2d give a diversity ordering MO>TR≈EM, which does not conform to ecologists' expectation.

**Figure 2:**
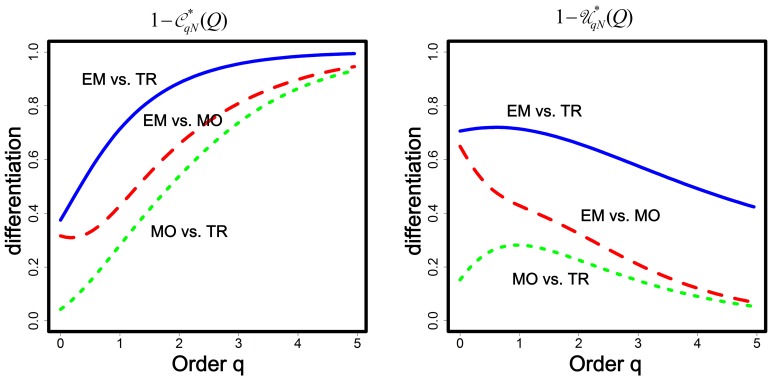
Differentiation profiles for the functional differentiation measures 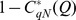 (left panel) and 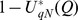 (right panel) as a function of order *q* for three pairs of habitats (EM vs. MO, EM vs. TR and MO vs. TR.)

In Fig. 2, we show the differentiation profiles of the two proposed measures 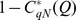 and 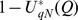 as a function of order *q* for *q* between 0 and 5. In Table 6, we compare various differentiation measures between any two habitats (EM vs. MO, EM vs. TR and MO vs. TR). In the same table, as we did in Table 4, we also show the differentiation values incorporating both abundance and function (*A*&*F*), function (*F*) only, and abundance (*A*) only. Table 6 reveals that in any pair of assemblages, we have a pattern similar to that in Table 4 for Matrix I. That is, our differentiation measures considering both (*A*&*F*) yield comparable results to those considering abundance only (*A*) for *q* = 0 and for *q* = 2, and yield very close results for *q* = 1. As with Example 1, this may be explained by the fact that the gamma quadratic entropy in each pair of assemblage is only slightly higher than the alpha quadratic entropy. The relative differences between gamma and alpha quadratic entropies is respectively 2.8%, 4.5% and 2.7% for EM vs. MO, EM vs. TR and MO vs. TR. Therefore, abundance is the major factor that determines the differentiation between any two habitats, implying that the four measures incorporating abundances with or without considering function exhibit very similar patterns in Fig. 1.

**Table 6:** Comparison of various differentiation measures for three pairs of habitats in the real data analysis based on abundance and function (*A&F*), on function (*F*) only, and abundance (*A*) only.

Measure	Order	EM vs. MO			EM vs. TR			MO vs. TR		
		*A&F*	*F*	*A* #	*A&F*	*F*	*A* #	*A&F*	*F*	*A* #
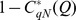	*q* = 0	0.316	0.316	0.392	0.375	0.375	0.457	0.043	0.043	0.062
	*q* = 1	0.428	---	0.427	0.714	---	0.721	0.282	---	0.278
	*q* = 2	0.658	---	0.573	0.885	---	0.854	0.539	---	0.457
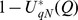	*q* = 0	0.649	0.649	0.564	0.706	0.706	0.628	0.152	0.152	0.118
	*q* = 1	0.428	---	0.427	0.714	---	0.721	0.282	---	0.278
	*q* = 2	0.324	---	0.401	0.659	---	0.746	0.226	---	0.296
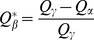	*q* = 2	0.028			0.042			0.026		
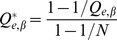	*q* = 2	0.066			0.102			0.067		
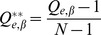	*q* = 2	0.034			0.054			0.035		

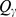
 = 0.550 and 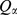 = 0.535 for the pair (EM, MO); 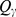 = 0.561, 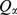 = 0.537 for the pair (EM, TR); 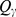 = 0.574, 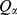 = 0.559 for the pair (MO, TR).

# Differentiation measures are the abundance-based local differentiation measure (1−*C_qN_*) and regional differentiation measure (1−*U_qN_*) obtained from partitioning Hill numbers [36];

--- No measures for *q* = 1 and *q* = 2 because species abundances are not considered for measures based on function (*F*) only.

Our proposed differentiation measures, 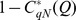 and 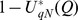 (Table 6 and Fig. 2) implies that EM vs. TR has the highest functional differentiation, MO vs. TR has the lowest differentiation, and EM vs. MO is somewhat in between for any fixed order *q* between 0 and 5. This pattern is anticipated. As discussed above, the vegetation within EM is composed by few specialized plants with similar ecological functions to adapt the extreme environmental stress. However, these traits are unique to species in EM when compared with species in the other two habitats. There are also fewer shared species between EM and TR (also EM and MO). In contrast, the vegetation in MO and TR is similarly diverse and most species in these two habitats are shared. These explain why MO vs. TR exhibits the lowest functional differentiation, whereas EM vs. TR (also EM vs. MO) exhibit higher functional differentiation.

Table 6 and Fig. 2 further reveal that the two measures 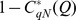 and 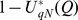 for the three pairs of habitats give moderate to high differentiation. For example, for *q* = 2, our differentiation measure 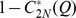 for the three pairs (EM vs. MO, EM vs. TR and MO vs. TR) is respectively 0.658, 0.885 and 0.539, and the corresponding differentiation measure 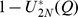 is respectively 0.324, 0.659 and 0.226. In sharp contrast, the three previous measures based on the quadratic entropy (Eqs. 2c, 2e and 2f) show substantially lower differentiation. For these data, the differentiation measure based on the additive decomposition of quadratic entropy (Eq. 2c) for EM vs. MO, EM vs. TR and MO vs. TR is respectively 0.028, 0.042 and 0.026. This wrongly implies substantially low differentiation between any two habitats. For the differentiation measure based on Eq. 2f are also low (0.034, 0.054 and 0.035). These values also give an unexpected ordering in that EM vs. MO exhibits the lowest functional differentiation, which is counter-intuitive. Similarly, the measure given in Eq. 2e gives a wrong ordering. All three examples demonstrate that our functional diversity measures and their associated differentiation measures yield the expected results and ecologically sensible interpretations.

## Conclusion and Discussion

We have extended ordinary Hill numbers to the distance-based functional Hill number 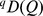 to take into account the pairwise functional distance between species (see Eq. 3, in units of effective number of equally abundant and equally distinct species). Here *Q* (Rao's quadratic entropy) plays an important indirect role, even though the measure *Q* itself cannot be directly used to measure functional diversity as noted by several authors [26], [35], [36], [39]. We have also proposed a class of mean functional diversity *^q^MD*(*Q*) = 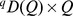; see Eq. 4a. The product of the functional Hill number and the mean functional diversity quantifies the (total) functional diversity *^q^FD*(*Q*) = 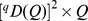, i.e., the effective total distance between species of the assemblage. See Fig. 1 for an example comparing ordinary Hill numbers and the three functional diversity measures. The three proposed measures quantify different aspects of species trait space. Our approach is valid not only for any symmetric distance matrices in ecology, but also for all types of symmetric matrices in other disciplines.

Since the pioneering work by MacArthur [40] and Hill [41], the replication principle has been identified as an essential property for characterizing abundance-based species diversity. As we reviewed in this paper, Hill numbers obey the replication principle. Hill numbers have been extended to phylogenetic Hill numbers (in units of “species equivalent”) and related branch diversity (in units of “branch length”); both satisfy a phylogenetic generalization of the replication principle [36], [45]. In this paper, we have proved that the functional Hill numbers (in units of “species equivalent”) and the mean functional diversity (in units of “functional distance”) both satisfy a functional version of the replication principle, and also proved that the functional diversity (in units of “functional distance”) satisfies a quadratic replication principle. Therefore, we think replication principle is an essential property for measures in units of species equivalents, but for other related measures this property may be valid (e.g., branch diversity, mean functional diversity), or may be replaced by a quadratic (or a power function of *N*) property; see [48] for a different perspective.

Recently, Chao et al. [50] integrated species diversity, phylogenetic diversity and functional diversity into a unified framework of “attribute diversity” based on Hill numbers. Both Leinster and Cobbold [75] and Scheiner [63] derived their integrative metrics under a framework of Hill numbers and their metrics are also in units of “species equivalents”. In Appendix S5, we provide detailed comparison to distinguish these two previous approaches from ours. Generally, we find that Leinster & Cobbold's measure may not be sensitive to species abundances when species similarity matrix is computed from species traits in functional analysis. If species similarity matrix deviates greatly from a naïve identity matrix, then their measure typically yields very low diversity values especially for assemblages with many species; this causes problems for the interpretation of “species equivalents” in their approach. Reeve et al. [88] recently proposed a diversity partition based on Leinster & Cobbold's measure. In the same Appendix, we show by an example that their gamma diversity may be less than their proposed alpha diversity even in equal weight case. Scheiner's approach and our measures have different meanings of “species equivalents” and thus quantify different aspects and properties of ecosystems. Scheiner's measure cannot be directly linked to most of the previous commonly used phylogenetic diversity (e.g., Faith's *PD*) and functional measures (e.g., *FAD*).

Except for Rao's quadratic entropy, the decomposition of other functional diversity measures is rarely discussed in the literature. In this paper, we have developed the decomposition of the proposed three functional diversity measures of any order *q*; see Table 2 for a summary. In the decomposition of each of the three measures, the alpha and beta components are unrelated (or independent), and thus each beta component measures pure functional differentiation among assemblages and can be transformed onto the range [0,1] to obtain the normalized distance-overlap measures (from the beta component based on the functional diversity) or species-overlap measures (from the beta components based on decomposing the functional Hill numbers and the mean functional diversity). In most applications, we recommend applying the distance-overlap measures (given in Table 3 for the two major classes of similarity measures). An important advantage of using the framework of Hill numbers is that there is a direct link between functional diversity measures and functional similarity (or differentiation) among assemblages. To convey the information about functional differentiation among multiple assemblages, we suggest plotting the differentiation profiles for two differentiation measures, 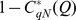 (from the perspective of a pair of local assemblages) and 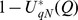 (from the perspective of the pooled assemblage) with respect to *q*; see Fig. 2 for examples. For the special case of *q* = 0, the measure 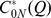 (Eq. 9b and Table 3) in terms of *FAD* represents the functional generalization of the Sørensen similarity index, and the measure 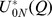 (Eq. 10b and Table 3) represents the functional generalization of the classic Jaccard similarity index. Conceptually different approach to diversity decomposition was proposed by Kosman [48].

Villéger et al. [20] proposed that trait-based functional diversity should include three aspects: functional richness, functional evenness and functional divergence. In ordinary Hill numbers, zero-order diversity represents species richness, and Hill numbers (or their transformations) of different orders can be used to construct various functional evenness measures as those proposed by Jost [89]. Jost used partitioning theory to derive Hill's useful class of evenness measures, the ratios of Hill numbers *^q^D* and species richness, *^q^D/S* for *q*>0, and he showed that the ratio of the logarithms of Hill numbers and logarithm of richness, log(*^q^D*)/log(*S*), expresses the corresponding relative evenness. Applying this idea to our framework, we can construct measures of functional richness and functional evenness based on any of our three functional diversity measures. For example, we can regard the effective total distance between species of order *q* = 0 as a measure of functional richness and use 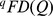/[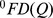] and log[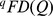]/log[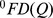] as measures of functional evenness.

Finally, we mention a potential application of our proposed measures. In genetics, the nucleotide diversity [90] is based on a nucleotide distance matrix. The elements of this distance matrix are obtained as the nucleotide differences between any two DNA sequences. Since our approach can be applied to any type of symmetric distance matrix, we expect our proposed measures would be useful in genetics. The nucleotide diversity in genetics is equivalent to Rao's quadratic entropy, and the corresponding differentiation measure *N_ST_* is identical to the traditional differentiation measure in Eq. 2c. We have shown in this paper by hypothetical and real examples that the measure in Eq. 2c does not provide legitimate measure of differentiation; see [36] for theoretical discussion. We have proposed unified and rigorous distance-overlap measures and their corresponding differentiation measures (in Table 3), and thus those measures merit investigation for applications to genetics.

## Supporting Information

Appendix S1 Some properties of the proposed functional diversity measures.
(PDF)

Appendix S2 Decomposition of the proposed functional diversity measures.
(PDF)

Appendix S3 Four classes of functional similarity/differentiation measures.
(PDF)

Appendix S4 Functional beta diversity and functional diversity excess lead to the same classes of similarity and differentiation measures.
(PDF)

Appendix S5 Supplementary examples and comparisons.
(PDF)

Appendix S6 Distance matrices used in Example 1 and Example 3.
(XLSX)
