# Correction: Distance-Based Functional Diversity Measures and Their Decomposition: A Framework Based on Hill Numbers

**DOI:** 10.1371/journal.pone.0113561

**Published:** 2014-11-14

**Authors:** The PLOS ONE Staff

There is an error in Table 1, “A framework for Hill numbers, functional Hill numbers, mean functional diversity and (total) functional diversity of a single assemblage.” Please see the corrected Table 1 here.

**Table 1:** A framework for Hill numbers, functional Hill numbers, mean functional diversity and (total) functional diversity of a single assemblage.

	Abundance vector/matrix	weights	*q*-th power sum ( *q*≠1)	Equating the two *q*-th power sums
**(1) Hill numbers**				
Actual assemblage	*S* species with relative abundance vector:	Unity weight for each species	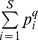	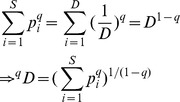
	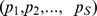	(1, 1, …., 1)		
Idealized reference assemblage	*D* equally-abundant species	Unity weight for each species	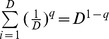	(Hill number of order *q*)
	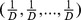	(1, 1, …., 1)		
**(2) Functional Hill number, mean functional diversity and (total) functional diversity**				
Actual assemblage	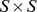 matrix of the product of relative abundances for pairs of species 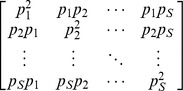	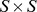 distance matrix as weight 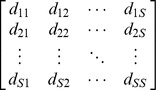	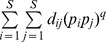	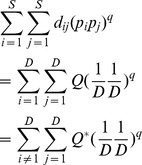
Idealized reference assemblage	*D×D* matrix of the product of equal relative abundances for pairs of species	*D×D* idealized distance matrix as weights	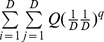 Or 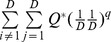	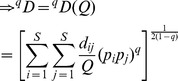
	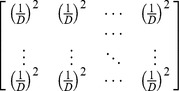	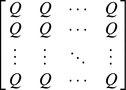		(Functional Hill number = number of rows or columns in th*e* idealized distance matrix) 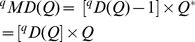
		or		
		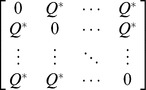		(Mean functional diversity = column/row sum in the idealized distance matrix)
		Q* = QD /(*D −*1)		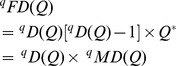
				(Total functional diversity = grand sum of the idealized distance matrix)

There are formatting errors in the Supporting Information files Appendix S1, Appendix S2, Appendix S3, Appendix S4, and Appendix S5. Please view the correct Appendix S1, Appendix S2, Appendix S3, Appendix S4, and Appendix S5 here.

## Supporting Information

Appendix S1 **Some properties of the proposed functional diversity measures.**
(PDF)

Appendix S2 **Decomposition of the proposed functional diversity measures.**
(PDF)

Appendix S3 **Four classes of functional similarity/differentiation measures.**
(PDF)

Appendix S4 **Functional beta diversity and functional diversity excess lead to the same classes of similarity and differentiation measures.**
(PDF)

Appendix S5 **Supplementary examples and comparisons.**
(PDF)
