# Supplementary material for: Distance-Based Functional Diversity Measures and Their Decomposition: A Framework Based on Hill Numbers
Source: PLoS One. 2014 Jul 7;9(7):e100014. doi: 10.1371/journal.pone.0100014 (PMC4085071; doi:10.1371/journal.pone.0100014)
Supplement: Appendix S2 — Decomposition of the proposed functional diversity measures. (PDF) [file pone.0100014.s002.pdf]

Supporting Information

Distance-based functional diversity measures and their decomposition: a framework based on Hill numbers

Chun-Huo Chiu and Anne Chao
Institute of Statistics, National Tsing Hua University, Hsin-Chu, Taiwan 30043

Appendix S2: Decomposition of the proposed functional diversity measures

When there are multiple assemblages defined by the investigator, as indicated in the main text, the functional Hill number ${}^{q}\mathcal{D}(Q)$ (Eq. 3 of the main text), mean functional diversity ${}^{q}MD(Q)$ (Eq. 4a) and total functional diversity ${}^{q}FD(Q)$ (Eq. 4b) of the pooled assemblage can each be decomposed into independent alpha and beta components using a derivation similar to that developed by Chiu et al. [1] for ordinary Hill numbers. The decomposition procedures of all three measures are generally parallel and interpretations are similar. A summary of the decomposition of all three measures along with their interpretations are given in Table 2 of the main text.

Since the decomposition procedures for the mean functional diversity and total functional diversity are better understood via partitioning the functional Hill numbers, we first present the details of decomposing the functional Hill numbers ${}^{q}\mathcal{D}(Q)$ and then apply the results to the total functional diversity ${}^{q}FD(Q)$. In this Appendix, we mainly focus on the decomposition of the functional Hill numbers and the total functional diversity.

The functional gamma Hill number is defined as the effective number of equally abundant and equally distinct species in the pooled assemblage, where species abundances are pooled over assemblages. As discussed in the main text, the species relative abundance set in the pooled assemblage can be expressed as $\{z_{i+}/z_{++};\ i = 1, 2,\ldots, S\}$. Thus, it follows from Eq. 3 of the main text that the functional gamma Hill number of order q is:

$$ {}^{q}\mathcal{D}_{\gamma}(Q) = \left[\sum_{i=1}^{S}\sum_{j=1}^{S}\frac{d_{ij}}{Q}\left(\frac{z_{i+}}{z_{++}}\frac{z_{j+}}{z_{++}}\right)^{q}\right]^{1/2(1-q)} \qquad q \ge 0,\ q \ne 1. \qquad \text{(B1)} $$

$$ {}^{1}\mathcal{D}_{\gamma}(Q) = \lim_{q\to 1} {}^{q}\mathcal{D}_{\gamma}(Q) = \exp\left[-\frac{1}{2}\sum_{i=1}^{S}\sum_{j=1}^{S}\frac{d_{ij}}{Q}\left(\frac{z_{i+}}{z_{++}}\frac{z_{j+}}{z_{++}}\right)\log\left(\frac{z_{i+}}{z_{++}}\frac{z_{j+}}{z_{++}}\right)\right]. $$

For a single assemblage, the Hill number of order q, or the "effective number of species", is defined as the number of equally abundant species that would be needed to give the same value of the diversity measure. We now extend this concept to functional alpha Hill number. Assume that there are N assemblages and there are S species in the pooled assemblage. For $i = 1, 2, \ldots, S$, $k = 1, 2, \ldots, N$, let z_{ik} denote the species abundance of the ith species in the kth assemblage. As discussed in the main text, the variable z_{ik} could be absolute abundance, relative abundances, incidences,

biomasses, or cover areas. We can formulate the S x N species abundance matrix $\mathbf{Z}$ as

$$\mathbf{Z} = \begin{bmatrix} z_{11} & z_{12} & \cdots & z_{1N} \\ z_{21} & \cdot & \cdots & \cdot \\ \cdot & \cdot & \cdots & \cdot \\ z_{S1} & z_{S2} & \cdots & z_{SN} \end{bmatrix}.$$

Let the kth column of $\mathbf{Z}$ be denoted by $\mathbf{z}_k = (z_{1k}, z_{2k}, ..., z_{Sk})^T$, (the super-script "$T$" denotes the "transpose" of a matrix) and this column denotes the species abundances of the kth assemblage. So the matrix $\mathbf{Z}$ can be denoted by ($\mathbf{z}_1, \mathbf{z}_2, \ldots, \mathbf{z}_N$). The matrix total is $z_{++} = \sum_{i=1}^{S} \sum_{j=1}^{N} z_{ij}$. Let d_{ij} denote the functional distance between the ith and the jth species, $d_{ij} \geq 0$, and $d_{ij} = d_{ji}$. Denote the S x S symmetric pairwise distance matrix by $\Delta = [d_{ij}]$.

Following Chiu, we define functional alpha Hill number as the effective number of equally abundant and equally distinct species per assemblage. Then the following derivation leads to a formula. Assume that each of the N assemblages is equivalent to an idealized reference assemblage which contains A species and all species are equally common with a constant abundance $\bar{a}$. That is, in the kth idealized assemblage, the abundance set can be expressed as an A x1 column vector $\mathbf{b}_k = (\bar{a}, \bar{a}, ..., \bar{a})^T$, $k = 1, 2, \ldots, N$. Whether there are shared species among these N idealized assemblages is not relevant because functional alpha Hill number is independent of shared information (i.e., the value is independent of the between-assemblage information). Without loss of generality, we assume the N idealized assemblages form an abundance matrix $\mathbf{B} = (\mathbf{b}_1, \mathbf{b}_2, \ldots, \mathbf{b}_N)$. A good feature of the idealized N-assemblage matrix $\mathbf{B}$ is that each assemblage has A equally abundant species and assemblage sizes are all equal.

Let Q be the quadratic entropy of the pooled assemblage of the actual assemblages, i.e., $Q = \sum_i \sum_j d_{ij} z_{i+} z_{j+} / z_{++}^2$. In the idealized assemblage, we have a constant distance Q for all species pairs; see Table 1 of the main text for illustration. The idealized distance matrix is denoted by $\Delta(Q)$.

The two matrices $\mathbf{Z}$ and $\mathbf{B}$ are "equivalent" in the sense that any q-th power sum ($q \geq 0$) of the elements of the matrix $\mathbf{Z} = (\mathbf{z}_1, \mathbf{z}_2, \ldots, \mathbf{z}_N)$ should give identical values when the same function is applied to the idealized reference matrix $\mathbf{B} = (\mathbf{b}_1, \mathbf{b}_2, \ldots, \mathbf{b}_N)$ with $\mathbf{b}_j = (\bar{a}, \bar{a}, ..., \bar{a})^T$ for all j. We consider the following two special functions:

(1) The sum of all elements in $\mathbf{Z}$ is thus equal to the corresponding sum in $\mathbf{B}$. This sum for $\mathbf{Z} = (\mathbf{z}_1, \mathbf{z}_2, \ldots, \mathbf{z}_N)$ is z_{++}, whereas the sum for $\mathbf{B} = (\mathbf{b}_1, \mathbf{b}_2, \ldots, \mathbf{b}_N)$ is $AN\bar{a}$ (since each column has A species and there are N columns with all elements being equal to $\bar{a}$). Thus we obtain $\bar{a} = z_{++} / (AN)$.

(2) Choose all possible combinations of any two columns of the matrix $\mathbf{Z}$ and form the weighted (by distance) q-th power sum:

$$h(\mathbf{Z},\Delta)=\sum_{k,m=1}^{N}\sum_{i,j=1}^{S}d_{ij}(z_{ik}z_{jm})^{q}\ .$$

Then the same function is applied to the simple reference assemblage to obtain

$$h(\mathbf{B},\varDelta(Q))=\sum_{k,m=1}^{N}\sum_{i,j=1}^{A}Q(\bar{a}\bar{a})^{q}=\sum_{k,m=1}^{N}\sum_{i,j=1}^{A}Q\left(\frac{z_{++}}{AN}\right)^{2q}=QA^{2(1-q)}N^{2(1-q)}(z_{++})^{2q}\,.$$

Equating these two functions shows that A (the proposed functional alpha Hill number) has the following form:

$$A={}^{q}\mathcal{D}_{\alpha}(Q)=\frac{1}{N}\left[\sum_{k,m=1}^{N}\sum_{i,j=1}^{S}\frac{d_{ij}}{Q}\left(\frac{z_{ik}}{z_{++}}\frac{z_{jm}}{z_{++}}\right)^{q}\right]^{\frac{1}{2(1-q)}}\qquad q\geq 0,\quad q\neq 1;\qquad \text{(B2)}$$

$${}^{1}\mathcal{D}_{\alpha}(Q)=\lim_{q\to 1}{}^{q}\mathcal{D}_{\alpha}(Q)=\frac{1}{N}\exp\left[-\frac{1}{2}\sum_{k,m=1}^{N}\sum_{i,j=1}^{S}\frac{d_{ij}}{Q}\left(\frac{z_{ik}}{z_{++}}\frac{z_{jm}}{z_{++}}\right)\log\left(\frac{z_{ik}}{z_{++}}\frac{z_{jm}}{z_{++}}\right)\right],\quad q=1.$$

This new alpha Hill number is interpreted as the "effective number of equally abundant and equally distinct species per assemblage". Note here in the above alpha formula, Q refers to the quadratic entropy of the pooled assemblage. Then based on a multiplicative partitioning, we define the functional beta Hill number of order q as the ratio:

$${}^{q}\mathcal{D}_{\beta}(Q)={}^{q}\mathcal{D}_{\gamma}(Q)/{}^{q}\mathcal{D}_{\alpha}(Q)\,.$$

This functional beta Hill number is interpreted as the effective number of equally large and completely distinct assemblages.

The above theory can be directly applied to decompose the (total) functional diversity ${}^{q}FD(Q)$. The functional gamma diversity is the effective total distance between species in the pooled assemblage with a constant distance Q for all species pairs, where Q denotes the quadratic entropy of the pooled assemblage. It follows from the derivation of the functional gamma Hill numbers that we have ${}^{q}FD_{\gamma}(Q)=Q\times[{}^{q}\mathcal{D}_{\gamma}(Q)]^{2}$, and formulas are given in Eqs. 6a and 6b in the main text. The functional alpha diversity is defined as the effective total distance between species of a pair of assemblages. Our above derivation implies that ${}^{q}FD_{\alpha}(Q)=Q\times[{}^{q}\mathcal{D}_{\alpha}(Q)]^{2}$. Substituting the functional alpha Hill number, we then obtain the formulas of the functional alpha diversity as shown in Eqs. 7a and 7b in the main text. The functional beta diversity based on a multiplicative rule is:

$${}^{q}FD_{\beta}(Q)=\frac{{}^{q}FD_{\gamma}(Q)}{{}^{q}FD_{\alpha}(Q)}=[{}^{q}\mathcal{D}_{\beta}(Q)]^{2}\,.$$

Similar gamma and alpha components for the mean functional diversity can be also derived respectively as ${}^{q}MD_{\gamma}(Q)=Q\times[{}^{q}\mathcal{D}_{\gamma}(Q)]$ and ${}^{q}MD_{\alpha}(Q)=Q\times[{}^{q}\mathcal{D}_{\alpha}(Q)]$; the corresponding beta component is

$${}^{q}MD_{\beta}(Q)=\frac{{}^{q}MD_{\gamma}(Q)}{{}^{q}MD_{\alpha}(Q)}={}^{q}\mathcal{D}_{\beta}(Q)\,.$$

See Table 2 of the main text for a summary.

In the following proposition, we prove for all $q \geq 0$ that the functional beta Hill number of order q is always between 1 and N regardless of the functional alpha Hill numbers. Also, the functional beta diversity of order q is always between 1 and N^2 regardless of the functional alpha diversity. Then the alpha and beta components obtained from decomposing each measure are unrelated (or independent).

For any arbitrary symmetric matrices and all orders of $q \geq 0$, when the number of assemblages, N, is fixed, the functional beta Hill number of order q is always in the range [1, N], i.e.,$1 \leq {}^q\mathcal{D}_\beta(Q) = {}^qMD_\beta(Q) \leq N$ for all $q \geq 0$. The functional beta diversity of order q is always in the range [1, N^2], i.e.,$1 \leq {}^qFD_\beta(Q) \leq N^2$ for all $q \geq 0$. These conclusions are based on the following proposition.

Proposition S2.1:

(a) For *all* $q \geq 0$, the functional alpha and gamma Hill numbers satisfy the following inequality:

$$ {}^q\mathcal{D}_\alpha(Q) \leq {}^q\mathcal{D}_\gamma(Q) \leq N[{}^q\mathcal{D}_\alpha(Q)], \tag{B3}$$

or equivalently,

$$[{}^q\mathcal{D}_\gamma(Q)]/N \leq {}^q\mathcal{D}_\alpha(Q) \leq {}^q\mathcal{D}_\gamma(Q). \tag{B4}$$

Similar inequalities are also valid for the mean functional alpha and gamma diversities.

(b) For *all* $q \geq 0$, the functional alpha and gamma diversities satisfy the following inequality:

$$ {}^qFD_\alpha(Q) \leq {}^qFD_\gamma(Q) \leq N^2[{}^qFD_\alpha(Q)], \tag{B5}$$

or equivalently,

$$ {}^qFD_\gamma(Q)/N^2 \leq {}^qFD_\alpha(Q) \leq {}^qFD_\gamma(Q). \tag{B6}$$

Proof: It is sufficient to prove (B3) and (B4) because (B5) and (B6) follow directly from (B3) and (B4). From Eqs. (B1) and (B2), the functional gamma and alpha Hill number for $q \neq 1$ is respectively

$$ {}^q\mathcal{D}_\gamma(Q) = \left[\sum_{i,j=1}^{S} \frac{d_{ij}}{Q}\left(\frac{z_{i+}}{z_{++}}\frac{z_{j+}}{z_{++}}\right)^q\right]^{\frac{1}{2(1-q)}} = \left[\sum_{i,j=1}^{S} \frac{d_{ij}}{Q}\left(\sum_{k=1}^{N}\frac{z_{ik}}{z_{++}}\sum_{m=1}^{N}\frac{z_{jm}}{z_{++}}\right)^q\right]^{\frac{1}{2(1-q)}},$$

and

$$ {}^q\mathcal{D}_\alpha(Q) = \frac{1}{N}\left[\sum_{k,m=1}^{N}\sum_{i,j=1}^{S} \frac{d_{ij}}{Q}\left(\frac{z_{ik}}{z_{++}}\frac{z_{jm}}{z_{++}}\right)^q\right]^{\frac{1}{2(1-q)}}.$$

We first prove ${}^q\mathcal{D}_\gamma(Q) \leq N \times [{}^q\mathcal{D}_\alpha(Q)]$. For $q > 1$, this conclusion follows directly from the following inequality:

$$[{}^q\mathcal{D}_\gamma(Q)]^{2(1-q)} = \sum_{i,j=1}^{S} \frac{d_{ij}}{Q}\left(\sum_{k=1}^{N}\frac{z_{ik}}{z_{++}}\sum_{m=1}^{N}\frac{z_{jm}}{z_{++}}\right)^q \geq \sum_{i,j=1}^{S}\frac{d_{ij}}{Q}\sum_{k,m=1}^{N}\left(\frac{z_{ik}}{z_{++}}\frac{z_{jm}}{z_{++}}\right)^q = [N\times{}^q\mathcal{D}_\alpha(T)]^{2(1-q)}.$$

Similarly, for $0 \leq q \leq 1$, the same conclusion follows from the following inequality:

$$[{}^q\mathcal{D}_\gamma(Q)]^{2(1-q)} = \sum_{i,j=1}^{S} \frac{d_{ij}}{Q}\left(\sum_{k=1}^{N}\frac{z_{ik}}{z_{++}}\sum_{m=1}^{N}\frac{z_{jm}}{z_{++}}\right)^q \leq \sum_{i,j=1}^{S}\frac{d_{ij}}{Q}\sum_{k,m=1}^{N}\left(\frac{z_{ik}}{z_{++}}\frac{z_{jm}}{z_{++}}\right)^q = [N\times{}^q\mathcal{D}_\alpha(Q)]^{2(1-q)}.$$

For $q = 1$, note that we have

$$\log{}^1\mathcal{D}_\gamma(Q) = -\frac{1}{2}\sum_{i=1}^{S}\sum_{j=1}^{S}\frac{d_{ij}}{Q}\left(\sum_{k=1}^{N}\frac{z_{ik}}{z_{++}}\sum_{m=1}^{N}\frac{z_{jm}}{z_{++}}\right)\log\left(\sum_{k=1}^{N}\frac{z_{ik}}{z_{++}}\sum_{m=1}^{N}\frac{z_{jm}}{z_{++}}\right),$$

and

$$\log[N\times{}^1\mathcal{D}_\alpha(Q)] = -\frac{1}{2}\sum_{k,m=1}^{N}\sum_{i,j=1}^{S}\frac{d_{ij}}{Q}\frac{z_{ik}}{z_{++}}\frac{z_{jm}}{z_{++}}\log\left(\frac{z_{ik}}{z_{++}}\frac{z_{jm}}{z_{++}}\right).$$

Since $\log\left(\sum_{k=1}^{N}\frac{z_{ik}}{z_{++}}\sum_{m=1}^{N}\frac{z_{jm}}{z_{++}}\right) \geq \log\left(\frac{z_{ik}}{z_{++}}\frac{z_{jm}}{z_{++}}\right)$, the proof is readily seen for $q = 1$.

To prove ${}^q\mathcal{D}_\gamma(Q) \geq {}^q\mathcal{D}_\alpha(Q)$, note that for $q > 1$, $f(x) = x^q$ is a convex function, so the Jensen inequality implies that for any species pair (i, j), we have

$$\left(\frac{1}{N^2}\sum_{k=1}^{N}\frac{z_{ik}}{z_{++}}\sum_{m=1}^{N}\frac{z_{jm}}{z_{++}}\right)^q \leq \frac{1}{N^2}\sum_{k=1}^{N}\sum_{m=1}^{N}\left(\frac{z_{ik}}{z_{++}}\frac{z_{jm}}{z_{++}}\right)^q.$$

That is,

$$\frac{1}{N^{2q}}\sum_{i,j=1}^{S}\frac{d_{ij}}{Q}\left(\sum_{k=1}^{N}\frac{z_{ik}}{z_{++}}\sum_{l=1}^{N}\frac{z_{jm}}{z_{++}}\right)^q \leq \frac{1}{N^2}\sum_{i,j=1}^{S}\frac{d_{ij}}{Q}\sum_{k=1}^{N}\sum_{m=1}^{N}\left(\frac{z_{ik}}{z_{++}}\frac{z_{jm}}{z_{++}}\right)^q,$$

which is equivalent to

$$N^{-2q}[{}^q\mathcal{D}_\gamma(Q)]^{2(1-q)} \leq N^{-2}[N\times{}^q\mathcal{D}_\alpha(Q)]^{2(1-q)},$$

yielding ${}^q\mathcal{D}_\alpha(Q) \leq {}^q\mathcal{D}_\gamma(Q)$ for $q > 1$.

For $0 \leq q < 1$, $f(x) = x^q$ is a concave function, so the Jensen inequality implies that for species pair (i, j), we have

$$\left(\frac{1}{N^2}\sum_{k=1}^{N}\frac{z_{ik}}{z_{++}}\sum_{m=1}^{N}\frac{z_{jm}}{z_{++}}\right)^q \geq \frac{1}{N^2}\sum_{k=1}^{N}\sum_{m=1}^{N}\left(\frac{z_{ik}}{z_{++}}\frac{z_{jm}}{z_{++}}\right)^q,$$

and thus

$$\frac{1}{N^{2q}}\sum_{i,j=1}^{S}\frac{d_{ij}}{Q}\left(\sum_{k=1}^{N}\frac{z_{ik}}{z_{++}}\sum_{l=1}^{N}\frac{z_{jm}}{z_{++}}\right)^q \geq \frac{1}{N^2}\sum_{i,j=1}^{S}\frac{d_{ij}}{Q}\sum_{k=1}^{N}\sum_{m=1}^{N}\left(\frac{z_{ik}}{z_{++}}\frac{z_{jm}}{z_{++}}\right)^q.$$

The above inequality is equivalent to

$$N^{-2q}[{}^qD_\gamma(Q)]^{2(1-q)} \ge N^{-2}[N \times {}^q\mathcal{D}_\alpha(Q)]^{2(1-q)},$$

which implies ${}^q\mathcal{D}_\gamma(Q) \le {}^q\mathcal{D}_\alpha(Q)$ for $0 \le q < 1$.

To prove ${}^q\mathcal{D}_\gamma(Q) \ge {}^q\mathcal{D}_\alpha(Q)$ for $q = 1$, note that $f(x) = -x\log x$ is a concave function. The Jensen inequality leads to

$$-\left(\frac{1}{N^2}\sum_{k,m=1}^{N}\frac{z_{ik}}{z_{++}}\frac{z_{jm}}{z_{++}}\right)\log\left(\frac{1}{N^2}\sum_{k,m=1}^{N}\frac{z_{ik}}{z_{++}}\frac{z_{jm}}{z_{++}}\right) \ge -\frac{1}{N^2}\sum_{k,m=1}^{N}\left(\frac{z_{ik}}{z_{++}}\frac{z_{jm}}{z_{++}}\right)\log\left(\frac{z_{ik}}{z_{++}}\frac{z_{jm}}{z_{++}}\right).$$

Then we have

$$-\sum_{i,j=1}^{S}\frac{d_{ij}}{Q}\left(\sum_{k,m=1}^{N}\frac{z_{ik}}{z_{++}}\frac{z_{jm}}{z_{++}}\right)\log\left(\frac{1}{N^2}\sum_{k,m=1}^{N}\frac{z_{ik}}{z_{++}}\frac{z_{jm}}{z_{++}}\right) \ge -\sum_{i,j=1}^{S}\frac{d_{ij}}{Q}\sum_{k,m=1}^{N}\left(\frac{z_{ik}}{z_{++}}\frac{z_{jm}}{z_{++}}\right)\log\left(\frac{z_{ik}}{z_{++}}\frac{z_{jm}}{z_{++}}\right).$$

Equivalently,

$$2\log N + 2\log {}^1\mathcal{D}_\gamma(Q) \ge 2\log N + 2\log {}^1\mathcal{D}_\alpha(Q).$$

Then the inequality ${}^1\mathcal{D}_\gamma(Q) \ge {}^1\mathcal{D}_\alpha(Q)$ is obtained.

Proposition S2.2 (A property of monotonicity used for Example 2 of the main text): Consider *N* assemblages indexed by 1, 2, …, *N*. Assume that species *a* is a non-shared species in an assemblage (say, Assemblage 1), i.e., species *a* does not exist in any other assemblages. Assume that species *b* is a non-shared species in a different assemblage (say, Assemblage 2). Let the functional distance between species *a* and species *b* be denoted by d_{ab}. Then the functional beta Hill number of order *q* is always a non-decreasing function with respect to d_{ab}. This implies that any differentiation measure based on the functional beta Hill number is also a non-decreasing function with respect to d_{ab}.

Proof: From the formulas of the functional alpha and gamma diversities, the functional beta diversity of order *q* is expressed as (see the main text for notation)

$${}^qFD_\beta(Q) = \frac{{}^qFD_\gamma(Q)}{{}^qFD_\alpha(Q)} = \left[\frac{\sum_{i,j=1}^{S} d_{ij}\left(\frac{z_{i+}z_{j+}}{Q z_{++}^2}\right)^q}{\frac{1}{N^{2(1-q)}}\sum_{k,m=1}^{N}\sum_{i,j=1}^{S} d_{ij}\left(\frac{z_{i+}z_{j+}}{Q z_{++}^2}\right)^q}\right]^{\frac{1}{(1-q)}}.$$

We can simplify the above formula as

$$[{}^qFD_\beta(Q)]^{1-q} = \frac{\sum_{i,j=1}^{S} d_{ij}(z_{i+}z_{j+})^q}{\frac{1}{N^{2(1-q)}}\sum_{k,m=1}^{N}\sum_{i,j=1}^{S} d_{ij}(z_{ik}z_{jm})^q} \equiv \frac{A}{B}.$$

We first prove the proposition for $q > 1$. For the non-shared species pair (a, b) with distance d_{ab} between these two species, we have

$$\frac{A}{B} = \frac{\sum_{i,j\neq(a,b)}^{S} d_{ij}(z_{i+}z_{j+})^q + d_{ab}(z_{a1}z_{b2})^q}{\frac{1}{N^{2(1-q)}}\sum_{k,m=1}^{N}\sum_{i,j\neq(a,b)}^{S} d_{ij}(z_{ik}z_{jm})^q + \frac{1}{N^{2(1-q)}} d_{ab}(z_{a1}z_{b2})^q} \quad . \qquad \text{(B7)}$$

The derivative of Eq. (B7) with respective to d_{ab} is

$$\left\{(z_{a1}z_{b2})^q\left[\frac{1}{N^{2(1-q)}}\sum_{k,m=1}^{N}\sum_{i,j\neq(a,b)}^{S} d_{ij}(z_{ik}z_{jm})^q\right] - \frac{1}{N^{2(1-q)}}(z_{a1}z_{b2})^q\left[\sum_{i,j\neq(a,b)}^{S} d_{ij}(z_{i+}z_{j+})^q\right]\right\}/B^2$$

$$= \frac{1}{N^{2(1-q)}}(z_{a1}z_{b2})^q\left\{\sum_{k,m=1}^{N}\sum_{i,j\neq(a,b)}^{S} d_{ij}(z_{ik}z_{jm})^q - \sum_{i,j\neq(a,b)}^{S} d_{ij}(z_{i+}z_{j+})^q\right\}/B^2 . \qquad \text{(B8)}$$

Since for $q > 1$ and for any distance between species i and j, we have

$$d_{ij}\left[\sum_{k,m=1}^{N}(z_{ik}z_{jm})^q\right] \le d_{ij}\left[\left(\sum_{k=1}^{N} z_{ik}\right)\left(\sum_{m=1}^{N} z_{jm}\right)\right]^q = d_{ij}(z_{i+}z_{j+})^q .$$

Thus, the derivative in Eq. (B8) must be non-positive. Therefore, $[{}^qFD_\beta(Q)]^{1-q}$ is non-increasing with d_{ab} for $q > 1$, implying that ${}^qFD_\beta(Q)$ is non-decreasing with d_{ab}. For $q < 1$, parallel steps also lead the same conclusion. The proof for $q = 1$ is direct.

Reference

1. Chiu C-H, Jost L, Chao A (2014) Phylogenetic beta diversity, similarity, and differentiation measures based on Hill numbers. Ecol Monogr 84: 21–44.
