# Supplementary material for: Distance-Based Functional Diversity Measures and Their Decomposition: A Framework Based on Hill Numbers
Source: PLoS One. 2014 Jul 7;9(7):e100014. doi: 10.1371/journal.pone.0100014 (PMC4085071; doi:10.1371/journal.pone.0100014)
Supplement: Appendix S3 — Four classes of functional similarity/differentiation measures. (PDF) [file pone.0100014.s003.pdf]

Supporting Information

Distance-based functional diversity measures and their decomposition: a framework based on Hill numbers

Chun-Huo Chiu and Anne Chao
Institute of Statistics, National Tsing Hua University, Hsin-Chu, Taiwan 30043

Appendix S3: Four classes of functional similarity/differentiation measures

As proved in Appendix S2, our functional beta Hill number ${}^{q}\mathcal{D}_{\beta}(Q)\,(={}^{q}MD_{\beta}(Q))$ always takes values between 1 and N (Proposition S2.1 in Appendix S2) and the functional beta diversity ${}^{q}FD_{\beta}(Q)$ always takes values between 1 and N^2. Thus both can be transformed onto [0, 1] to obtain normalized similarity and differentiation measures among assemblages. The similarity measures based on ${}^{q}\mathcal{D}_{\beta}(Q)$ quantify *species-overlap* from different perspectives, whereas the similarity measures based on ${}^{q}FD_{\beta}(Q)$ quantify *distance-overlap* from different perspectives. In most applications, we suggest using the distance-overlap (or similarity) measures and their corresponding differentiation measures. In the main text, we have briefly introduced the two major classes of normalized distance-overlap measures; see Table 3 of the main text. Here we provide more details along with interpretations for all four classes of distance-overlap measures.

(A) Similarity/differentiation measures based on the functional beta diversity (summarized in Table 3 of the main text)

(1) A class of local (functional) distance-overlap measures from the perspective of a pair of local assemblages

$$\mathcal{C}^{*}_{qN}(Q)=\frac{N^{2(1-q)}-[{}^{q}FD_{\beta}(Q)]^{1-q}}{N^{2(1-q)}-1}. \qquad \text{(C1)}$$

This measure gives the effective average proportion of the species pairwise distances in a pair of local assemblages that are shared with all other pairs of assemblages.

(1a) $q = 0$

We first give the special case of $q = 0$ to intuitively explain its properties: since ${}^{0}FD_{\gamma}(Q)=FAD_{\gamma}$ and ${}^{0}FD_{\alpha}(Q)=FAD_{pair}/N^2$, the measure $\mathcal{C}^{*}_{0N}(Q)$ reduces to

$$\mathcal{C}^{*}_{0N}(Q)=\frac{(FAD_{pair}-FAD_{\gamma})/(N^2-1)}{FAD_{pair}/N^2}, \qquad \text{(C2)}$$

where FAD_{γ} denotes the sum of pairwise distances in the pooled assemblage, and FAD_{pair} is

the sum of *FAD*s over all possible pairs of assemblages (there are N^2 pairs of assemblages). As discussed in the text, the measure $\mathcal{C}^*_{0N}(Q)$ thus quantifies the proportion of repeated distances in a pair of local assemblages. Its interpretation is conceptually analogous to the classic Sørensen similarity index and can be regarded as an extension of the classic Sørensen index to functional similarity. It is referred to as "func-Sørensen" in Table 3 of the main text. For the special case of $N = 2$, let the index set Ω_1 denote the species in Assemblage I, Ω_2 denote the species in Assemblage II, and Ω_{12} denote the shared species (species in both assemblages). Then we can rewrite Eq. (C2) as

$$\mathcal{C}^*_{02}(Q) = \frac{\left(2\sum_{i\in\Omega_1}\sum_{j\in\Omega_{12}} d_{ij} + 2\sum_{i\in\Omega_2}\sum_{j\in\Omega_{12}} d_{ij} - \sum_{i\in\Omega_{12}}\sum_{j\in\Omega_{12}} d_{ij}\right)/3}{\left(\sum_{i\in\Omega_1}\sum_{j\in\Omega_1} d_{ij} + 2\sum_{i\in\Omega_1}\sum_{j\in\Omega_2} d_{ij} + \sum_{i\in\Omega_2}\sum_{j\in\Omega_2} d_{ij}\right)/4}. \qquad \text{(C3)}$$

In this expression, the denominator is the alpha functional diversity of order 0, which is the average of *FAD*s over all four assemblage pairs (I, I), (I, II), (II, I) and (II, II); the numerator is the average of all repeated distances in the $N^2-1= 3$ pairs of assemblages (excluding the assemblage-pair in which a distance is first counted).

(1b) q =1

Letting q in Eq. (C1) tend to 1, we have

$$\mathcal{C}^*_{1N}(Q) = 1 - \frac{\log {}^1FD_\gamma(Q) - \log {}^1FD_\alpha(Q)}{2\log N}. \qquad \text{(C4)}$$

We refer to it as the N-assemblage "func-Horn" distance-overlap measure because when all distances are identical and (z_{ik}) represents species relative abundance within each assemblage, it reduces to the classical Horn measure. [1].

(1c) q =2

The general formula for $\mathcal{C}^*_{2N}(Q)$ shown in Table 3 of the main text is:

$$\mathcal{C}^*_{2N}(Q) = \frac{\sum_{i,j}^{S} d_{ij}\sum_{k,m}^{N}\left[\left(\frac{z_{i+}z_{j+}}{N}\right)^2 - (z_{ik}z_{jm})^2\right]}{(N^2-1)\sum_{i,j}^{S}\sum_{k,m}^{N} d_{ij}(z_{ik}z_{jm})^2}.$$

Here we give the formula for $N = 2$ to interpret the measure. Let $h_{11} = \frac{z_{i1}}{z_{++}}\frac{z_{j1}}{z_{++}}$ be the product of relative abundances of species pair (i, j) in the assemblage-pair (I, I), and a similar interpretation pertains to the notation $h_{12} = \frac{z_{i1}}{z_{++}}\frac{z_{j2}}{z_{++}}$, $h_{21} = \frac{z_{i2}}{z_{++}}\frac{z_{j1}}{z_{++}}$, $h_{22} = \frac{z_{i2}}{z_{++}}\frac{z_{j2}}{z_{++}}$. Then the measure $\mathcal{C}^*_{2N}(Q)$ for the special case of $N = 2$ reduces to

$$\mathcal{C}_{22}^{*}(Q)=\frac{\sum_{i,j}^{S} d_{ij} 2[h_{11}h_{12}+h_{11}h_{21}+h_{11}h_{22}+h_{12}h_{21}+h_{12}h_{22}+h_{21}h_{22}]/12}{\sum_{i,j}^{S} d_{ij}[h_{11}h_{11}+h_{12}h_{12}+h_{21}h_{21}+h_{22}h_{22}]/4}. \quad \text{(C5)}$$

It is called "func-Morisita-Horn" similarity measure because the concept is generally similar to the classic Morisita-Horn measure [2]. The denominator in Eq. (C5) represents the weighted (by abundances) average of distances for any two identical pairs of assemblages (there are four identical pairs, i.e., (I, I) vs. (I, I), (I, II) vs. (I, II), (II, I) vs. (II, I) and (II, II) vs. (II, II)).The numerator in Eq. (C5) represents the weighted (by abundances) average of *shared distances* for any two non-identical pairs of assemblages (there are 12 pairs, i.e., (I, I) vs. (I, II) (same as (I, II) vs. (I, I)), (I, I) vs. (II, I) (same as (II, I) vs. (I, I)), …, (II, I) vs. (II, II) (same as (II, II) vs. (II, I).) This interpretation extends the formulation of Chao et al. [2] for the classic Morisita-Horn measure to its functional version. The difference is that here we consider "assemblage-pairs" rather than "individual assemblage" as in the classical measure.

(1d) A general order of $q \geq 0$

Here the sense of "effective" is similar to that for the abundance-based local overlap measure C_{qN} [1,2], but "N assemblages" should be replaced with "N^2 pairs of assemblages", and "species" should be replaced by the unit of "distance". The meaning of "effective" average proportion for a value of $\mathcal{C}_{qN}^{*}(Q)=p$ is described as follows. We can construct a set of N^2 idealized assemblage-pairs: the total species pairwise distance of the assemblage-pair is ${}^{q}FD_{\alpha}(Q)$ units of "distance", with exactly ${}^{q}FD_{\alpha}(Q)\times p$ units of "distance" shared by all of them, and the remaining units of "distance" of each assemblage-pair not shared by any other assemblage-pairs. In the pooled assemblage, the gamma functional diversity is ${}^{q}FD_{\gamma}(Q)$ units of "distance". Then the $\mathcal{C}_{qN}^{*}(Q)$ measure of the actual set of N^2 assemblage-pairs is the same as that of this set of idealized assemblage-pairs. This gives a simple set of idealized assemblage-pairs to intuitively understand the interpretation of the measure $\mathcal{C}_{qN}^{*}(Q)$.

(2) A class of regional (functional) distance-overlap measures in the pooled assemblage

$$\mathcal{U}_{qN}^{*}(Q)=\frac{[1/{}^{q}FD_{\beta}(Q)]^{1-q}-(1/N)^{2(1-q)}}{1-(1/N)^{2(1-q)}}. \quad \text{(C6)}$$

This measure quantifies the effective proportion of the species pairwise distances in the pooled assemblage that are shared with all pairs of local assemblages.

(2a) $q = 0$

For the special case of $q = 0$, the measure $\mathcal{U}_{0N}^{*}(Q)$ reduces to

$$\mathcal{U}_{0N}^{*}(Q) = \frac{(FAD_{pair} - FAD_{\gamma})/(N^2-1)}{FAD_{\gamma}}. \quad \text{(C7)}$$

The interpretation is similar to the measure $\mathcal{C}_{0N}^{*}(Q)$ except that it is normalized by the gamma *FAD*. Thus, our index can be regarded as an extension of the Jaccard index to functional similarity. So this measure $\mathcal{U}_{0N}^{*}(Q)$ is referred to as the *N*-assemblage "func-Jaccard" in Table 3 of the main text. For the special case of $N = 2$, we have

$$\mathcal{U}_{02}^{*}(Q) = \frac{\left(2\sum_{i\in\Omega_1}\sum_{j\in\Omega_{12}} d_{ij} + 2\sum_{i\in\Omega_2}\sum_{j\in\Omega_{12}} d_{ij} - \sum_{i\in\Omega_{12}}\sum_{j\in\Omega_{12}} d_{ij}\right)/3}{\sum_{i\in\Omega}\sum_{j\in\Omega} d_{ij}}, \quad \text{(C8)}$$

Where Ω_1, Ω_2 and Ω_{12} are defined earlier and Ω denotes the species in the pooled assemblage. Comparing this expression with $\mathcal{C}_{02}^{*}(Q)$ in Eq. (C3), we see the numerators of the two measures are identical, but here it is normalized by FAD_{γ} in the denominator (i.e., from a regional view).

(2b) *q* = 1

Letting *q* in Eq. (C6) tend to 1, we obtain the same formula as the *N*-assemblage local distance-overlap measure $\mathcal{C}_{1N}^{*}(Q)$. That is, $\mathcal{U}_{1N}^{*}(Q) = \mathcal{C}_{1N}^{*}(Q)$. Hence the measure $\mathcal{U}_{1N}^{*}(Q)$ is also called "func-Horn" measure in Table 3 of the main text.

(2c) *q* = 2

The general formula for $\mathcal{U}_{2N}^{*}(Q)$ shown in Table 3 of the main text is:

$$\mathcal{U}_{2N}^{*}(Q) = \frac{\sum_{i,j}^{S} d_{ij} \sum_{k,m}^{N}\left[\left(\frac{z_{i+}z_{j+}}{N}\right)^2 - (z_{ik}z_{jm})^2\right]}{(1-1/N^2)\sum_{i,j}^{S} d_{ij}(z_{i+}z_{j+})^2}.$$

Here we give the formula for $N = 2$ to interpret the measure:

$$\mathcal{U}_{22}^{*}(Q) = \frac{\sum_{i,j}^{S} d_{ij}\, 2\left[h_{11}h_{12} + h_{11}h_{21} + h_{11}h_{22} + h_{12}h_{21} + h_{12}h_{22} + h_{21}h_{22}\right]/12}{\sum_{i,j}^{S} d_{ij}(\bar{p}_{i+}\bar{p}_{j+})^2}. \quad \text{(C9)}$$

The numerator is the same as that in Eq. (C5), but here it is from a regional perspective. So it is referred to as "func-regional-overlap" measure in Table 3 of the main text.

(2d) A general order of $q \geq 0$

Here the sense of "effective" is similar to that for the abundance-based measure U_{qN} [1], but "N assemblages" should be replaced with "N^2 pairs of assemblages", and "species" should be replaced by the unit of "distance". The meaning of "effective" proportion for the measure $\mathcal{U}^*_{qN}(Q)$ is slightly different from that for the measure $\mathcal{C}^*_{qN}(Q)$. When $\mathcal{U}^*_{qN}(Q) = u$, consider the following set of N idealized assemblages: in the pooled assemblage, the total species pairwise distance is ${}^qFD_\gamma(Q)$ units of "distance", with exactly ${}^qFD_\gamma(Q) \times u$ units of "distance" shared by all N^2 assemblage-pairs, and the remaining ${}^qFD_\gamma(Q) \times (1-u)$ units of "distance" evenly distributed in N^2 assemblage-pairs. In each of the N idealized assemblages, the functional diversity is ${}^qFD_\alpha(Q)$ units of "distance". Then the $\mathcal{U}^*_{qN}(Q)$ measure of the set of actual assemblages is the same as that of this set of idealized assemblage-pairs. Thus, we can intuitively understand the interpretation of the measure $\mathcal{U}^*_{qN}(Q)$.

(3) A class of functional distance-homogeneity measures

$$\mathcal{S}^*_{qN}(Q) = \frac{1/[{}^qFD_\beta(Q)] - 1/N^2}{1 - 1/N^2}.$$

For $q = 0$, it is the N-assemblage "func-Jaccard" measure $\mathcal{U}^*_{0N}(Q)$. For $q = 2$, this measure is identical to $\mathcal{C}^*_{2N}(Q)$, the "func-Morisita-Horn" measure. Thus, we have $\mathcal{S}^*_{0N}(Q) = \mathcal{U}^*_{0N}(Q)$ and $\mathcal{S}^*_{2N}(Q) = \mathcal{C}^*_{2N}(Q)$. However, for $q = 1$, this measure does not reduce to the "func-Horn" measure.

(4) A class of the complement of distance-turnover rate

$$\mathcal{V}^*_{qN}(Q) = \frac{N^2 - {}^qFD_\beta(Q)}{N^2 - 1}.$$

The corresponding differentiation measure $[{}^qFD_\beta(Q) - 1]/(N^2 - 1)$ quantifies distance-turnover rate per assemblage pair. When $q = 0$, the measure $\mathcal{V}^*_{0N}(Q)$ is identical to the "func-Sørensen" measure. For $q = 2$, this measure is identical to $\mathcal{U}^*_{2N}(Q)$, the "func-regional-overlap" measure. That is, we have $\mathcal{V}^*_{0N}(Q) = \mathcal{C}^*_{0N}(Q)$ and $\mathcal{V}^*_{2N}(Q) = \mathcal{U}^*_{2N}(Q)$. However, for $q = 1$, this measure does not reduce to the "func-Horn" measure.

(B) Similarity/differentiation measures based on the functional beta Hill numbers (Table S3.1)

(1) A class of local (functional) species-overlap measures

$$\mathcal{C}_{qN}(Q) = \frac{N^{1-q} - [{}^q\mathcal{D}_\beta(Q)]^{1-q}}{N^{1-q} - 1}. \qquad \text{(C10)}$$

This measure is interpreted as the effective average proportion of the species in an individual

assemblage that are shared with all other assemblages. The corresponding differentiation measure $1-\mathcal{C}_{qN}(Q)$ quantifies the effective average proportion of the total non-shared species in an individual assemblage.

(1a) *q* = 0

We first write the formula for the special case of $q = 0$ to intuitively understand the measure:

$$\mathcal{C}_{0N}(Q)=\frac{N-N(FAD_{\gamma}/FAD_{pair})^{1/2}}{N-1}=\frac{(FAD_{pair})^{1/2}-(FAD_{\gamma})^{1/2}}{(N-1)(FAD_{pair})^{1/2}/N}. \qquad \text{(C11)}$$

In the special case that all species are equally distinct, we have $FAD_{\gamma}=S^2\times Q$ and $FAD_{pair}=(N\bar{S})^2\times Q$, where S is the species richness in the pooled assemblage and $\bar{S}$ is the average species richness per assemblage. Consequently, the measure $\mathcal{C}_{0N}(Q)$ reduces to the classic measure C_{0N} (N-assemblage Sørensen similarity measure [2,3]), i.e., $C_{0N}=\dfrac{(N\bar{S}-S)}{(N-1)\bar{S}}$. From this view, the measure $\mathcal{C}_{0N}(Q)$ is referred to as the "func-Sørensen (species-overlap)" measure in Table S3.1. We add "species-overlap" in order to distinguish it from "func-Sørensen" (distance-overlap) based on the functional beta diversity in Table 3 of the main text.

(1b) *q* = 1

For $q = 1$, we have the following formula:

$$\mathcal{C}_{1N}(Q)=1-\frac{\log[{}^1\mathcal{D}_{\gamma}(Q)]-\log[{}^1\mathcal{D}_{\alpha}(Q)]}{\log N}.$$

This measure is referred to as the "func-Horn" measure because $\mathcal{C}^*_{1N}(Q)=\mathcal{C}_{1N}(Q)$ and $\mathcal{C}^*_{1N}(Q)$ is called "func-Horn" in Table 3 of the main text. This indicates that for $q = 1$, the local distance- and species-overlap measures are identical.

(1c) *q* = 2

For $q = 2$, the general formula of $\mathcal{C}_{2N}(Q)$ can be expressed as

$$\mathcal{C}_{2N}(Q)=\frac{\left(\sum_{i,j}d_{ij}(z_{i+}z_{j+})^2\right)^{1/2}-\left(\sum_{k,m}\sum_{i,j}d_{ij}(z_{ik}z_{jm})^2\right)^{1/2}}{(N-1)\left(\sum_{k,m}\sum_{i,j}d_{ij}(z_{ik}z_{jm})^2\right)^{1/2}}.$$

This measure is called as the "func-Morisita-Horn (species-overlap)" measure because when all species are equally distinct and the data (z_{ik}) represents species relative abundance within each assemblage, it reduces to the classic N-assemblage Morisita-Horn overlap measure [2,3].

(1d) A general order of *q* ≥ 0

The measure $\mathcal{C}_{qN}(Q)$ is interpreted as the effective average proportion of the species in an individual assemblage that are shared with all other assemblages. The interpretation of "effective" for this measure is a direct extension of the classic measure C_{qN} discussed in Chao et al. [2,3] and Chiu et al. [1]. When $\mathcal{C}_{qN}(Q) = p$, We can construct a set of N idealized assemblages: each of the N assemblages has ${}^q\mathcal{D}_\alpha(Q)$ equally abundant and functionally equally distinct species (all pairwise distances are identical), with exactly ${}^q\mathcal{D}_\alpha(Q) \times p$ species shared by all of them, and the remaining species of each assemblage not shared by any other assemblages. In the pooled assemblage, the gamma functional Hill number is ${}^q\mathcal{D}_\gamma(Q)$. Then the $\mathcal{C}_{qN}(Q)$ measure of the actual set of N assemblages is the same as that of this set of idealized assemblages. This gives a simple set of idealized assemblages to intuitively understand the interpretation of the measure $\mathcal{C}_{qN}(Q)$.

(2) A class of regional (functional) species-overlap measures

$$\mathcal{U}_{qN}(Q) = \frac{[1/{}^q\mathcal{D}_\beta(Q)]^{1-q} - (1/N)^{1-q}}{1-(1/N)^{1-q}}. \qquad \text{(C12)}$$

This class of measures differs from the local functional species-overlap measures by taking a regional perspective. They give the effective proportion of the species in the pooled assemblage that are shared with all other assemblages. The corresponding differentiation measure $1-\mathcal{U}_{qN}(Q)$ quantifies the effective proportion of the total non-shared species in the pooled assemblage.

(2a) *q* = 0

For $q = 0$, the measure reduces to

$$\mathcal{U}_{0N}(Q) = \frac{(FAD_{pair}/FAD_\gamma)^{1/2} - 1}{N-1} = \frac{(FAD_{pair})^{1/2} - (FAD_\gamma)^{1/2}}{(N-1)(FAD_\gamma)^{1/2}}. \qquad \text{(C13)}$$

In the special case that all species are equally distinct, $\mathcal{U}_{0N}(Q)$ reduces to the classic measure U_{0N} (N-assemblage Jaccard similarity measure [1]), i.e., $U_{0N} = \dfrac{(N\bar{S} - S)}{(N-1)S}$; see Table S3.1 at the end of this appendix. So the measure $\mathcal{U}_{0N}(Q)$ is referred to as the N-assemblage "func-Jaccard (species-overlap)" measure in Table S3.1. We add "species-overlap" in order to distinguish it from "func-Jaccard" (distance-overlap) measure based on the functional beta diversity in Table 3 of the main text.

(2b) For $q = 1$, this measure is identical to the N-assemblage "func-Horn" overlap measure $\mathcal{C}_{1N}(Q)$. So this measure has unique feature that both ${}^qFD_\beta(Q)$ and ${}^q\mathcal{D}_\beta(Q)$ lead to the same local and regional overlap measures, i.e., $\mathcal{C}^*_{1N}(Q) = \mathcal{C}_{1N}(Q) = \mathcal{U}^*_{1N}(Q) = \mathcal{U}_{1N}(Q)$.

(2c) For q = 2, the general formula can be expressed as

$$\mathcal{U}_{2N}(Q)=\frac{\left(\sum_{i,j} d_{ij}(z_{i+}z_{j+})^2\right)^{1/2}-\left(\sum_{k,m}\sum_{i,j} d_{ij}(z_{ik}z_{jm})^2\right)^{1/2}}{(1-1/N)\left(\sum_{i,j} d_{ij}(z_{i+}z_{j+})^2\right)^{1/2}}.$$

The numerator is the same as that of the measure $\mathcal{C}_{2N}(Q)$, but the denominator is from a regional view. So it is referred to as "func-regional-overlap (species-overlap)" in Table S3.1.

(2d) A general order of $q \geq 0$

The measure $\mathcal{U}_{qN}(Q)$ is interpreted as the effective average proportion of the species in the pooled assemblage that are shared with all other assemblages. The meaning of "effective" for this class is slightly different from that for $\mathcal{C}_{qN}(Q)$. When $\mathcal{U}_{qN}(Q)=u$, consider the following set of idealized assemblages: in the pooled assemblage, we have ${}^q\mathcal{D}_\gamma(Q)$ equally abundant and equally distinct species, with exactly ${}^q\mathcal{D}_\gamma(Q)\times u$ species shared by all N assemblages, and the remaining ${}^q\mathcal{D}_\gamma(Q)\times(1-u)$ species evenly distributed in N assemblages. In each of the N idealized assemblages, the effective functional Hill number is ${}^q\mathcal{D}_\alpha(Q)$. Then the measure $\mathcal{U}_{qN}(Q)$ of the set of actual assemblages is the same as that of this set of idealized assemblages. This gives a simple set of idealized assemblages to intuitively understand the interpretation of the measure $\mathcal{U}_{qN}(Q)$.

<u>(3) A class of functional species-homogeneity measures</u>

$$\mathcal{S}_{qN}(Q)=\frac{1/{}^q\mathcal{D}_\beta(Q)-1/N}{1-1/N}.$$

For q = 0, this measure is the "func-Jaccard (species-overlap)" measure $\mathcal{U}_{0N}(Q)$. For q = 2, this measure is identical to $\mathcal{C}_{2N}(Q)$, the "func-Morisita-Horn (species-overlap)" similarity measure. Thus, we have $\mathcal{S}_{0N}(Q)=\mathcal{U}_{0N}(Q)$ and $\mathcal{S}_{2N}(Q)=\mathcal{C}_{2N}(Q)$. However, for q = 1, this measure does not reduce to the "func-Horn" overlap measure.

<u>(4) A class of the complement of functional species-turnover rate</u>

$$\mathcal{V}_{qN}(Q)=\frac{N-{}^q\mathcal{D}_\beta(Q)}{N-1}.$$

The corresponding differentiation measure $1-\mathcal{V}_{qN}(Q)$ is the normalized functional species-turnover rate per assemblage. When q = 0, the measure is identical to the "func-Sørensen (species-overlap)" measure. For q = 2, this measure is identical to $\mathcal{U}_{2N}(Q)$, the "func-regional-overlap (species-overlap)" measure. That is, we have $\mathcal{V}_{0N}(Q)=\mathcal{C}_{0N}(Q)$ and

$\mathcal{V}_{2N}(Q) = \mathcal{U}_{2N}(Q)$. However, for $q = 1$, this measure does not reduce to the "func-Horn" overlap measure.

Table S3.1. Two classes of functional species-overlap (or similarity) measures and their special cases. The corresponding differentiation measures are the one-complements of the overlap measures. In the second column, "taxonomic" measures refer to the special case that all species are equally distinct as in the classic abundance-based measures. (The indices *i* and *j* are used to identify species, $i, j = 1, 2, \ldots, S$, and the indices *k* and *m* are used to identify assemblages, $k, m = 1, 2, \ldots, N$.)

Order	Measure	Local species-overlap $\mathcal{C}_{qN}(Q) = \dfrac{N^{1-q} - [{}^q\mathcal{D}_\beta(Q)]^{1-q}}{N^{1-q} - 1}$	Regional species-overlap $\mathcal{U}_{qN}(Q) = \dfrac{[1/{}^q\mathcal{D}_\beta(Q)]^{1-q} - (1/N)^{1-q}}{1 - (1/N)^{1-q}}$
$q = 0$	Functional	Func-Sørensen (species-overlap) $\dfrac{N - N(FAD_\gamma)^{1/2}/(FAD_{pair})^{1/2}}{N-1}$	Func-Jaccard (species-overlap) $\dfrac{(FAD_{pair})^{1/2}/(FAD_\gamma)^{1/2} - 1}{N-1}$
	Taxonomic	Classic Sørensen $\dfrac{N - S\ /\bar{S}}{N-1}$	Classic Jaccard $\dfrac{N\bar{S}/S - 1}{N-1}$
$q = 1$	Functional	Func-Horn $1 - \dfrac{\log[{}^1\mathcal{D}_\gamma(Q)] - \log[{}^1\mathcal{D}_\alpha(Q)]}{\log N}$	
	Taxonomic	Classic Horn $\dfrac{H_{Sh,\alpha} - H_{Sh,\gamma} - \sum_{k=1}^{N} \frac{z_{+k}}{z_{++}} \log\left(\frac{z_{+k}}{z_{++}}\right)}{\log N}$; $1 - \dfrac{H_{Sh,\gamma} - H_{Sh,\alpha}}{\log N}$ (if $z_{+k}=1$, $z_{++}=N$)	
$q = 2$	Functional	Func-Morisita-Horn (species-overlap) $\dfrac{\left(\sum_{i,j} d_{ij}(z_{i+}z_{j+})^2\right)^{1/2} - \left(\sum_{k,m}\sum_{i,j} d_{ij}(z_{ik}z_{jm})^2\right)^{1/2}}{(N-1)\left(\sum_{k,m}\sum_{i,j} d_{ij}(z_{ik}z_{jm})^2\right)^{1/2}}$	Func-regional-overlap (species-overlap) $\dfrac{\left(\sum_{i,j} d_{ij}(z_{i+}z_{j+})^2\right)^{1/2} - \left(\sum_{k,m}\sum_{i,j} d_{ij}(z_{ik}z_{jm})^2\right)^{1/2}}{(1-1/N)\left(\sum_{i,j} d_{ij}(z_{i+}z_{j+})^2\right)^{1/2}}$
	Taxonomic	Classic Morisita-Horn $1 - \dfrac{\sum_{i=1}^{S}\sum_{m>k}(z_{im} - z_{ik})^2}{(N-1)\sum_{i=1}^{S}\sum_{k=1}^{N} z_{ik}^2}$; $1 - \dfrac{H_{GS,\gamma} - H_{GS,\alpha}}{(1-1/N)(1-H_{GS,\alpha})}$ $(if\ z_{+k} = 1, z_{++} = N)$	Classic regional-overlap $1 - \dfrac{\sum_{i=1}^{S}\sum_{m>k}(z_{im} - z_{ik})^2}{(N-1)\sum_{i=1}^{S} z_{i+}^2}$; $1 - \dfrac{H_{GS,\gamma} - H_{GS,\alpha}}{(N-1)(1-H_{GS,\gamma})}$ $(if\ z_{+k} = 1, z_{++} = N)$

Notes:

(1) z_{ik} = the abundance of the *i*th species in the *k*th assemblage, $z_{+k} = \sum_{i=1}^{S} z_{ik}$, $z_{i+} = \sum_{k=1}^{N} z_{ik}$, and $z_{++} = \sum_{i=1}^{S}\sum_{k=1}^{N} z_{ik}$; see the main text for details. If z_{ik} represents species relative abundance, then in all formulas we have $z_{+k} = 1$, $z_{++} = N$.

(2) $q = 0$. FAD_γ = sum of the pairwise distances between species in the pooled assemblage; FAD_{pair} = sum of *FAD*s over all possible pairs of assemblages (there are N^2 pairs of assemblages). S = species richness in the pooled assemblage. $\bar{S}$ = average species richness per

assemblage.

(3) $q = 1$. $H_{Sh,\gamma}, H_{Sh,\alpha}$ = gamma and alpha Shannon entropy.

(4) $q = 2$. $H_{GS,\gamma}, H_{GS,\alpha}$ = gamma and alpha Gini-Simpson index.

References

1. Chiu C-H, Jost L, Chao A (2014) Phylogenetic beta diversity, similarity, and differentiation measures based on Hill numbers. Ecol Monogr 84: 21–44.
2. Chao A, Jost L, Chiang SC, Jiang Y-H, Chazdon RL (2008) A two-stage probabilistic approach to multiple-community similarity indices. Biometrics 64: 1178-1186.
3. Chao A, Chiu C-H, Hsieh TC (2012) Proposing a resolution to debates on diversity partitioning. Ecology 93: 2037–2051.
