# Supplementary material for: Distance-Based Functional Diversity Measures and Their Decomposition: A Framework Based on Hill Numbers
Source: PLoS One. 2014 Jul 7;9(7):e100014. doi: 10.1371/journal.pone.0100014 (PMC4085071; doi:10.1371/journal.pone.0100014)
Supplement: Appendix S5 — Supplementary examples and comparisons. (PDF) [file pone.0100014.s005.pdf]

Supporting Information

Distance-based functional diversity measures and their decomposition: a framework based on Hill numbers

Chun-Huo Chiu and Anne Chao
Institute of Statistics, National Tsing Hua University, Hsin-Chu, Taiwan 30043

Appendix S5: Supplementary examples and comparisons

(All equation numbers refer to those in the main text.)

Example: A simple functional distance matrix to show the counter-intuitive behavior of the traditional differentiation measure based on an additive partitioning of the quadratic entropy

Consider two hypothetical completely distinct assemblages (i.e. no shared species) each with S equally common species. Assume that the functional distance is a constant d for distinct-species pairs and 0 for same-species pairs. We first apply Rao's quadratic entropy to this simple distance matrix. The quadratic entropy Q of each assemblage is $d(1-1/S)$. This would be the alpha quadratic entropy Q_α of the assemblages. For any fixed d, the alpha value will be high (i.e., tends to the maximum possible value of d) if and only if species richness S is high. In the pooled assemblage, there are $2S$ equally common species with a constant functional distance d for distinct-species pairs and 0 for same-species pairs, so the gamma quadratic entropy of the pooled assemblage is $Q_\gamma = d[1-1/(2S)]$.

The traditional differentiation measure based on the additive partitioning is $Q_\beta^* = (Q_\gamma - Q_\alpha)/Q_\gamma$; see Eq. 2c. Thus, the differentiation measure is $[d/(2S)]/\,[d - d/(2S)] = 1/(2S-1)$, a number that approaches zero (wrongly indicating that there is almost no differentiation) when alpha is high (equivalently, when S is high), even though the two assemblages are completely distinct. Chiu et al. [1] proved that when the alpha quadratic entropy is high, the additive differentiation measure based on the quadratic entropy always tends to zero for any assemblages, not only for simple completely distinct assemblages, but also for more complicated assemblages and real data; see Example 3 of the main text.

The resolution in Eq. 2d of the main text works because this simple distance matrix is ultrametric. The effective number of species for the alpha diversity is $1/(1-Q_\alpha/d) = S$ and the effective number of species for the gamma diversity is $1/(1-Q_\gamma/d) = 2S$. Thus the multiplicative beta based on these effective measures is 2 and the two transformed differentiation measures in Eqs. 2e and 2f are both unity, which correctly indicates the differentiation attains the maximum. Our proposed normalized functional differentiation measures $1-\mathcal{C}_{qN}(Q)$ and $1-\mathcal{U}_{qN}(Q)$ (Table 3 of the main text) are always 1 for any q, any richness S, and any functional distance d. For this example, the correct answer is unequivocal: the two completely distinct assemblages in this simple case should attain the maximum differentiation of unity. This example shows that the traditional

measures based on the additive partitioning of the quadratic entropy cannot work properly even for this simple example.

Two supplementary cases for Example 1 of the main text

In Example 1 of the main text, we specifically consider the special case that all species in the two assemblages are equally abundant for illustrative purposes. Here we append two more cases that species abundances are heterogeneous in both assemblages.

Case 1: Shared species are abundant species

In Assemblage I, species abundances are 0.01 (for Species 1–Species 5), 0.02 (for Species 6–Species 10), and 0.085 (for Species 11–Species 20);
In Assemblage II, species abundances are 0.05 (for Species 9, 10, 11–18), 0.075 (for Species 19, 20, 21–23), and 0.025 (for Species 24 –Species 28);
There are 12 shared species (Species 9, 10, 11– 20), and a total of 28 species in the pooled assemblage.

Table S5.1. Comparison of various differentiation measures for Matrix I (with Q_γ = 0.463, Q_α = 0.462) and Matrix II (with Q_γ = 0.118, Q_α = 0.102) based on abundance and function (*A&F*), on function (*F*) only, and abundance (*A*) only.

Measure	Order	Matrix I			Matrix II		
		A&F	*F*	$A^{\#}$	*A&F*	*F*	$A^{\#}$
$1-\mathcal{C}^*_{qN}(Q)$	$q=0$	0.324	0.324	0.4	0.579	0.579	0.4
	$q=1$	0.271	---	0.267	0.365	---	0.267
	$q=2$	0.322	---	0.256	0.342	---	0.256
$1-\mathcal{U}^*_{qN}(Q)$	$q=0$	0.658	0.658	0.571	0.846	0.846	0.571
	$q=1$	0.271	---	0.267	0.365	---	0.267
	$q=2$	0.106	---	0.147	0.115	---	0.147
$Q^*_\beta = \dfrac{Q_\gamma - Q_\alpha}{Q_\gamma}$	$q=2$	0.003			0.134		
$Q^*_{e,\beta} = \dfrac{1-1/Q_{e,\beta}}{1-1/N}$	$q=2$	0.006			0.035		
$Q^{**}_{e,\beta} = \dfrac{Q_{e,\beta}-1}{N-1}$	$q=2$	0.003			0.018		

Differentiation measures are the abundance-based local differentiation measure (1–C_{qN}) and regional differentiation measure (1–U_{qN}) obtained from partitioning Hill numbers [1];
--- No measures for q = 1 and q = 2 because species abundances are not considered for measures based on function (*F*) only.

Case 2: Shared species are rare species

In Assemblage I, species abundances are 0.085 (for Species 1–Species 10), 0.01 (for Species 11–Species 15), and 0.002 (for Species 16–Species 20);
In Assemblage II, species abundances are 0.025 (for Species 9, 10, 11– 13), 0.05 (for Species 14, 15, 16 –23), and 0.075 (for Species 24 –species 28);
There are 12 shared species (Species 9, 10, 11– 20), and a total of 28 species in the pooled assemblage.

Table S5.2. Comparison of various differentiation measures for Matrix I (with Q_γ = 0.480, Q_α = 0.475) and Matrix II (with Q_γ = 0.244, Q_α = 0.098) based on abundance and function (*A&F*), on function (*F*) only, and abundance (*A*) only.

Measure	Order	Matrix I			Matrix II		
		A&F	*F*	*A*#	*A&F*	*F*	*A*#
$1-\mathcal{C}^*_{qN}(Q)$	q = 0#	0.324	0.324	0.4	0.579	0.579	0.4
	q = 1	0.681	---	0.680	0.864	---	0.680
	q = 2	0.879	---	0.832	0.957	---	0.832
$1-\mathcal{U}^*_{qN}(Q)$	q = 0	0.658	0.658	0.571	0.846	0.846	0.571
	q = 1	0.681	---	0.680	0.864	---	0.680
	q = 2	0.646	---	0.712	0.848	---	0.712
$Q^*_\beta = 1 - Q_\alpha / Q_\gamma$	q = 2	0.010			0.598		
$Q^*_{e,\beta} = \dfrac{1-1/Q_{e,\beta}}{1-1/N}$	q = 2	0.022			0.325		
$Q^{**}_{e,\beta} = \dfrac{Q_{e,\beta}-1}{N-1}$	q = 2	0.011			0.194		

Differentiation measures are the abundance-based local differentiation measure ($1–C_{qN}$) and regional differentiation measure ($1–U_{qN}$) obtained from partitioning Hill numbers [1];
--- No measures for q = 1 and q = 2 because species abundances are not considered for measures based on function (*F*) only.

A supplementary case for Example 2 of the main text

In Example 2 of the main text, we consider the special case that the two focal assemblages are completely distinct (no species shared, and thus no pairwise distances shared). Here we present similar results for a case that there are shared species between the two assemblages.

Table S5.3. Comparison of various differentiation measures between two assemblages for an ultramteric distance matrix (Case I below) and a non-ultrametric distance matrix (Case II below). Assume the two assemblages are not completely distinct. There are three equally common species (*a*, *b*, *c*) in the first assemblage, and three equally common species (*b*, *c*, *d*) in the second assemblage. In the pooled assemblage, there are four species (*a*, *b*, *c*, *d*) with relative abundances (0.167, 0.333, 0.333, 0.167). As explained in the text, we expect that the differentiation for Case II should not be lower than that for Case I.

Case I: An ultrametric distance matrix for four species (*a*, *b*, *c*, *d*) with $Q_\gamma = 0.122$, $Q_\alpha = 0.111$.

$$\begin{bmatrix} 0 & 0.1 & 0.2 & 0.2 \\ 0.1 & 0 & 0.2 & 0.2 \\ 0.2 & 0.2 & 0 & 0.1 \\ 0.2 & 0.2 & 0.1 & 0 \end{bmatrix}$$

Case II: A non-ultrametric distance matrix for four species (*a*, *b*, *c*, *d*) with $Q_\gamma = 0.161$, $Q_\alpha = 0.111$.

$$\begin{bmatrix} 0 & 0.1 & 0.2 & 0.9 \\ 0.1 & 0 & 0.2 & 0.2 \\ 0.2 & 0.2 & 0 & 0.1 \\ 0.9 & 0.2 & 0.1 & 0 \end{bmatrix}$$

Measure	Order *q*	Ultrametric distance matrix (Case I)	Non-ultrametric distance matrix (Case II)
$1-\mathcal{C}^*_{qN}(Q)$	$q = 0$	0.273	0.448
	$q = 1$	0.364	0.517
	$q = 2$	0.455	0.586
$1-\mathcal{U}^*_{qN}(Q)$	$q = 0$	0.600	0.765
	$q = 1$	0.364	0.517
	$q = 2$	0.172	0.262
$Q^*_\beta = \dfrac{Q_\gamma - Q_\alpha}{Q_\gamma}$	$q = 2$	0.091	0.310
$Q^*_{e,\beta} = \dfrac{1-1/Q_{e,\beta}}{1-1/N}$	$q = 2$	0.250	0.127
$Q^{**}_{e,\beta} = \dfrac{Q_{e,\beta}-1}{N-1}$	$q = 2$	0.143	0.068

Comparison of our framework with Leinster & Cobbold (2012) approach

Leinster & Cobbold [2] derived a parametric class of measures sensitive to species similarity based on a framework of Hill numbers. The similarity may be based on phylogeny, ecosystem function, or any other species character. We find that their measure (referred to as the LC measure hereafter) may not be sensitive to species abundances when species similarity matrix is computed from species traits in functional analysis. When species similarity matrix deviates greatly from a naïve identity matrix, their measure typically yields very low diversity values especially for assemblages with many species; this causes problems for the interpretation of "species equivalents" in their approach. Note that in the bottom right panel of Fig. 3 of [2], as q varies between 0 and 5, their measure for a non-naive similarity matrix decreases from 1.27 to 1.25 for Case "TS1" with ~250 species and decreases from 1.25 to 1.22 for Case "TS3" with ~ 200 species, as shown in the bottom left panel of their Fig. 3. This reveals that the LC measure hardly varies with the order q for the two cases considered in their Fig. 3. We thus computed several other real examples to see whether the LC measure generally exhibits a similar pattern. We describe two typical examples to show our findings.

We applied the LC measure to the artificial data (Example 2 of the main text) and the real data (Example 3 of the main text), so that readers can make comparisons. In Example 2, there are 20 equally abundant species in each of the two focal assemblages, and 12 species are shared. Two simulated distance matrices with all distances between 0 and 1 (Matrix I and Matrix II, displayed in Appendix S6) are considered; see the main text. In Example 3, the full data contain a total of 43 vascular plant species collected three fore-dune habitats: embryo dunes (EM; 17 species), mobile dunes (MO; 39 species) and transition dunes (TR; 42 species). The species relative abundances are provided in Table S5.1 of this appendix). The distance matrix for 43 species is displayed in Appendix S6. All species distances (between 0 and 1) are obtained from the Gower mixed-variables coefficient of distance. Since the LC measure is based on species similarity matrix, we considered two types of similarity metrics computed from the distance matrices: (i) the one-complement of each distance; (ii) the transformation exp(–d) of each distance d. The plot of the LC measure with respect to the order q for the two types of similarity metrics is given in the following figure for the two examples.

(a) The Leinster & Cobbold (LC) measure for two matrices based on artificial data in Example 2 of the main text

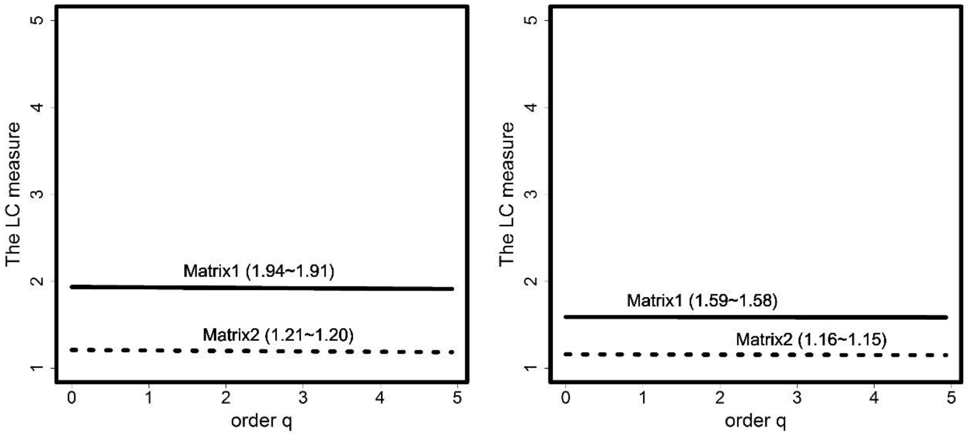

(b) The Leinster & Cobbold (LC) measure for three habitats (EM, MO and TR) based on real data in Example 3 of the main text

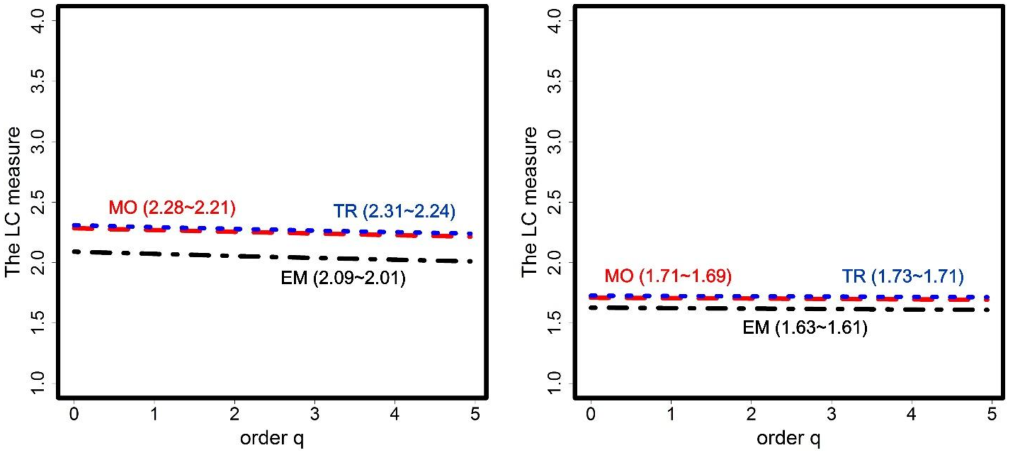

Figure S5.1. Diversity profiles as a function of the order q ($0 \le q \le 5$) of the Leinster and Cobbold (LC) measure for two examples. In the left panels, the similarity is defined as the one-complement of a distance. In the right panels, similarity is defined as exp(–*d*) of a distance *d*. The "range" in the plots and in the following captions refers to the range of the LC measure when q is increased from 0 to 5.

(Upper left panel) Matrix I (Solid line, in range: 1.94~ 1.91) and Matrix II (dotted line, in range: 1.21~1.20).

(Upper right panel) Matrix I (Solid line, in range: 1.59~ 1.58) and Matrix II (dotted line, in range: 1.16~1.15).

(Lower left panel) EM Habitat (black line, in range: 2.09~ 2.01), MO Habitat (red line, in range: 2.28~ 2.21) and TR Habitat (blue line, in range: 2.31~ 2.24).

(Lower right panel) EM Habitat (black line, in range: 1.63~ 1.61), MO Habitat (red line, in range: 1.71~ 1.69) and TR Habitat (blue line, in range: 1.73~ 1.71).

The above plots reveal that the LC measure takes values in a very narrow range and this range hardly changes for the two different types of similarity matrices. Since the order q controls the measure's sensitivity to species relative abundances and a larger value of q place progressively more weight on common species, these plots in Figure S5.1 demonstrate that the LC measure may not be sensitive to the species abundances. We have found similar patterns for many other data sets. A related question is thus how to interpret the magnitude and "effective numbers" of the LC measure.

Recently, Reeve et al. [3] proposed formulas for the alpha, beta and gamma diversities based on the LC measure and on the decomposition framework of ordinary Hill numbers. Here we consider the simplest equal weight case and apply Reeve et al. formulas to a simple similarity matrix. Consider two communities each with four species (1, 2, 3, 4). The relative abundances of the four species in community I are (0.98, 0.003, 0.001, 0.016). The relative abundances of the same four species for community II are (0.90, 0.009, 0.082, 0.009). Assume that the pairwise similarity for the four species is given by this matrix:

$$\begin{bmatrix} 1 & 0.9 & 0.9 & 0 \\ 0.9 & 1 & 0 & 0 \\ 0.9 & 0 & 1 & 0.9 \\ 0 & 0 & 0.9 & 1 \end{bmatrix}$$

Then we obtain the following alpha, beta and gamma diversity for four values of q:

Order	Gamma	Alpha
q = 0	1.2605	1.5375
q = 0.5	1.1120	1.1570
q = 1	1.0611	1.0679
q = 2	1.0343	1.0345

For this case, the gamma LC measure is less than the alpha LC measure not only for the four specific values of q in the above table but also for all values of $q \geq 0$. This situation violates the necessary condition that alpha must always be less than or equal to gamma.

Leinster & Cobbold ([2], p. 478) indicated that their metric has close connections with the phylogenetic indices of Faith [4] and Chao et al. [5]. This may be a misleading statement. In their Appendix, Leinster & Cobbold demonstrated that their formula could include Faith's *PD* and Chao et al. [5] phylogenetic Hill number only for a *particular* constructed similarity matrix (possibly non-symmetric) and a special set of species abundances. Note that their particular similarity matrix for *species* depends on species relative abundance. Thus, when two communities have the same set of species with different sets of species abundances, the corresponding particular similarity matrices are then different. Even within a single community, if two samples result in different

species abundances, then LC's particular similarity matrices are different. Thus, the "connection" between their metric and Chao et al. measure is only based on an uninterpretable similarity matrix. A useful "connection" between two measures should be based on any *given* matrix in a broad class, not just for a single particular constructed matrix.

For any given ultrametric tree, we can divide each species pairwise phylogenetic distance by the tree depth so that all distances are scaled to be in the range [0, 1]. When the similarity between any two species is defined as the one-complement of the scaled distance, the LC measure for q = 2 reduces to the Chao et al.'s phylogenetic Hill number of the same order. This is the only general connection that we have found between the LC measure and Chao et al. [5] phylogenetic Hill numbers if the similarity matrix is not a naive identity matrix.

Comparison of our framework with Scheiner (2012) approach

Scheiner [6] proposed a metric that integrates abundance, phylogeny and function based on a framework of Hill numbers. Our framework (Chao et al. [5] for phylogenetic diversity, and this paper for functional diversity) is also based on Hill numbers. However, the two approaches are completely different. In this section, we describe our fundamental concept and discuss the differences between our framework and Scheiner's approach.

The basic difference

The major difference lies in the interpretation of Hill numbers. In Scheiner's approach, the ordinary Hill numbers are interpreted as the *variability* in relative abundances among species. Based on this approach, Scheiner's phylogenetic diversity quantifies the variability of proportional phylogenetic divergences of species, and his functional diversity quantifies the variability of proportional functional distinctiveness.

Our interpretation of Hill numbers is different. The fundamental concept in our approach is based on the fact that there is a *unique* idealized assemblage with equally abundant species so that the actual assemblage and this idealized assemblage have the same diversity of order q. Thus, our extension to phylogenetic diversity and functional diversity leads to completely different measures as briefly described as follows.

(i) Phylogenetic diversity measures

Our phylogenetic Hill number (or mean phylogenetic diversity) of order q, denoted by ${}^q\overline{D}(T)$, is the effective number of equally abundant and equally phylogenetically distinct species with a constant branch length $\overline{T}$ from the root node. Here $\overline{T}$ denotes the abundance-weighted mean distance from a tip node to the root node; see Fig. 1 of Chao et al. [5] for the definition of $\overline{T}$. For an ultrametric tree with tree length T, then $\overline{T}$ reduces to the tree length T, and the measure is simply denoted by ${}^q\overline{D}(T)$. Generally, if ${}^q\overline{D}(T) = z$, then the phylogenetic Hill number of the assemblage is the same as the diversity of an idealized assemblage consisting of z equally abundant and (phylogenetically) equally distinct lineages all with branch length $\overline{T}$ from the root node. The basic concept is that there exists a *unique* idealized assemblage with equally abundant and equally

distinct species so that the actual assemblage and this idealized assemblage have the same diversity of order q.

The phylogenetic Hill number (in units of "species equivalent") does not incorporate information about the actual length of the phylogenetic tree because it is independent of the scale of lineage lengths. To incorporate the units of lineage length, we also proposed the phylogenetic diversity ${}^{q}PD(\bar{T})$ (in units of "lineage length"), ${}^{q}PD(\bar{T}) = \bar{T} \times [{}^{q}\bar{D}(\bar{T})]$. Thus, we not only have a measure in units of "species equivalents" but also a measure in units of "lineage length". This is more useful biologically since it expresses the amount of evolutionary history in the tree (with branches weighted by the size of their contribution to the present-day assemblage), and is also fruitful mathematically because we then can link our measures to Faith *PD* (for $q = 0$), phylogenetic entropy [7] (for $q = 1$), and Rao's quadratic entropy (for $q = 2$). Scheiner's measure cannot be linked to the phylogenetic entropy, nor to Rao's quadratic entropy.

(ii) Functional diversity measures

Our functional Hill number denoted by ${}^{q}\mathscr{D}(Q)$ (see Table 1 of the main text) is interpreted as "the effective number of equally abundant and (functionally) equally distinct species". Thus if ${}^{q}\mathscr{D}(Q) = v$, then the functional Hill number of order q of the actual assemblage is the same as that of an idealized assemblage having v equally abundant and equally distinct species with a constant distance Q for all species pairs. Our concept for functional diversity is based on the fact that there exists a *unique* idealized assemblage with equally abundant and equally distinct species so that the actual assemblage and this idealized assemblage have the same diversity of order q.

As with our phylogenetic Hill numbers, the functional Hill numbers ${}^{q}\mathscr{D}(Q)$ (in units of "species equivalent") are scale-free, so they need to be converted to our functional diversity ${}^{q}FD(Q)$ (the effective total functional distance between species), defined as ${}^{q}FD(Q) = Q[{}^{q}\mathscr{D}(Q)]^2$. Thus, we can link our measures to *FAD* (for $q = 0$), and to the weighted Gini-Simpson index (for $q = 2$) defined by Guiasu & Guiasu [8,9]; see the main text for details. To our knowledge, Scheiner's metric cannot be linked to these two previous measures.

<u>Different meanings of "species equivalents"</u>

Scheiner's integrated metric and our phylogenetic (and functional) Hill number are both in units of "effective number of species" or "species equivalents", and the "species equivalent" in both approaches is interpreted as the equally abundant and equally distinct species. However, the definition of "equally distinct" diverges between Scheiner's approach and ours. We use a simple example to illustrate the two different meanings.

(i) Phylogenetic diversity measures

Consider the following three assemblages with ultrametric cladograms. Each assemblage includes four species and the tree depth is $T = 8$ units. The number along each branch segment denotes the length of that branch. For each assemblage, we assume that all four species are equally abundant.

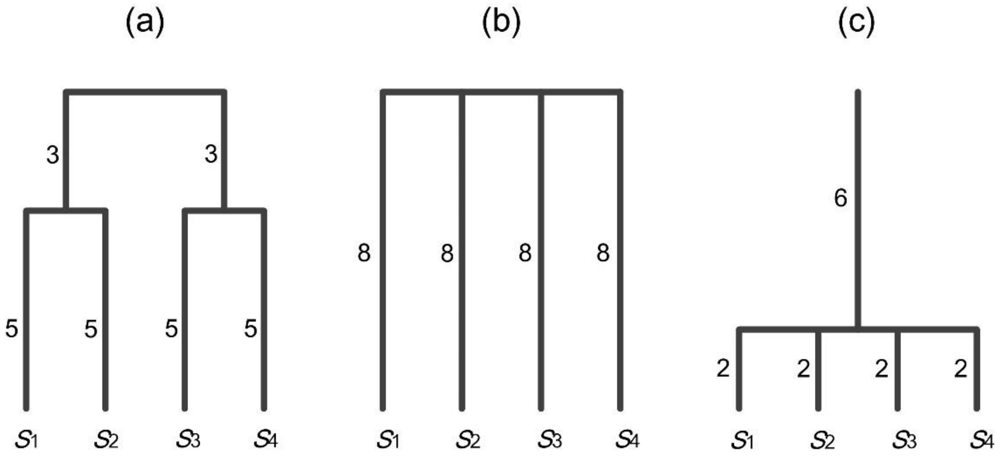

For the above three cladograms with $T = 8$ units, we give our phylogenetic Hill numbers ${}^{q}\overline{D}(T)$ (in units of "species equivalents"), phylogenetic diversity ${}^{q}PD(T)$ (in units of "lineage length") and Scheiner's phylogenetic diversity in the following table.

Measure	Order	Assemblage/Cladogram		
		(a)	(b)	(c)
Chao et al. (2010)	$q = 0$	26	32	14
phylogenetic	$q = 1$	24.78	32	11.31
diversity ${}^{q}PD(T)$	$q = 2$	23.27	32	9.85
Chao et al. (2010)	$q = 0$	3.25	4	1.75
phylogenetic Hill	$q = 1$	3.08	4	1.41
number ${}^{q}\overline{D}(T)$	$q = 2$	2.91	4	1.23
Scheiner (2012)	$q = 0$	4	4	4
phylogenetic	$q = 1$	4	4	4
diversity	$q = 2$	4	4	4

All the above three cladograms have the same proportional divergences as defined by Scheiner. For any $q \geq 0$, his phylogenetic diversity which quantifies the variability of proportional phylogenetic divergences of species thus yields four equally distinct species for all three assemblages. When his measure takes a maximum value of four, the assemblage may correspond to the four equally abundant species in cladograms (a), (b), (c) or any other symmetric or balanced cladograms. This explains why Scheiner ([6], p. 1195) indicated that his metric is a measure of tree "symmetry" or "balance". His measure cannot distinguish the difference among the three assemblages, and thus "species equivalents" does not have a unique reference assemblage.

Our phylogenetic measures ${}^{q}\overline{D}(\overline{T})$ and ${}^{q}PD(\overline{T})$ both satisfy the "weak monotonicity" property [5]. This property requires that if a newly added rarest species is maximally distinct from all other

species in the assemblage, then a phylogenetic measure should not decrease. However, Scheiner's phylogenetic diversity measure does not satisfy this property. Note that for $q > 0$, if such a species is added to the assemblage (a) or (c) in the above figure, that tree becomes non-symmetric, implying a possible decline in a measure of symmetry.

Note that in Scheiner's measure, "equally distinct" means species are equally divergent from the age of the root node. Our definition of "equally distinct" implies that *any two species must have a constant phylogenetic distance of* $\bar{T}$ (*or T* in an ultrametric tree), or equivalently, *all branch lengths must be equal to* $\bar{T}$ (*or T*) as cladogram (b) given above. For cladogram (a), the distance between Species 1 and Species 2 is 5 units whereas the distance between Species 1 and Species 3 is 8 units, so the species in cladogram (a) are not "equally distinct" in our perspective. Similarly, the four species in cladogram (c) are not "equally distinct" with branch lengths of 8 units either. Only cladogram (b) is the unique idealized assemblages with all species being "equally distinct" with all branch lengths of 8 units. Thus, for the "effective number of species" in our phylogenetic diversity measure, there exists a unique reference assemblage so that the actual assemblage and this idealized assemblage have the same diversity of order *q*. For example, in the special case of $q = 0$, the phylogenetic Hill number of cladogram (c) is 1.75. Then this means the zero-order diversity of the assemblage is the same as an idealized assemblage with 1.75 equally abundant species with a constant branch length of 8 units, i.e., the cladogram of the idealized reference assemblage is like cladogram (b) but only with 1.75 species.

The above table reveals that when diversity is based on our phylogenetic Hill number ${}^q\bar{D}(T)$ and phylogenetic diversity ${}^qPD(T)$, the three assemblages for any *q* have consistent ordering: (b) > (a) > (c), whereas Scheiner's measure shows (a) = (b) = (c). Ecologists may use this example to choose the measure to be used in their analysis.

(ii) Functional diversity measures

We use a simple example to compare the difference between our functional diversity measures and Scheiner's approach. Consider the following example: In Assemblage A, all species are equally distinct with species pairwise distance $d_{ij} = 0.1$ units; In Assemblage B, all species are equally distinct with $d_{ij} = 0.9$ units. Scheiner's functional diversity quantifying the variability of functional distinctiveness will give the same functional diversity for these two assemblages. Yet, from our approach, there are *S* species with a constant distance of 0.1 for all species pairs in Assemblage A, and our functional diversity (i.e., effective total distance) between species is S^2 x 0.1. For Assemblage B, there are *S* species with a constant distance of 0.9 for all species pairs, and the functional diversity is S^2 x 0.9. The effective numbers of species are the same for the two assemblages, but the total distance between species for the two assemblages are different. Thus, Scheiner's measure loses the information about the magnitude of species pairwise distances, which we think is important to characterize distance-based traits diversity.

Table S5.4. Species identities and relative abundances in three dune habitats: upper beach and embryo dunes (EM, 17 species), mobile dunes (MO, 39 species) and transition dunes (TR, 42 species). A relative abundance of 0 for a species in a habitat means that the species does not exist in that habitat. See the main text for data description and details.

Species	EM	MO	TR
Ammophila arenaria	0	0.118	0.006
Anthemis maritima	0.024	0.132	0.046
Asparagus acutifolius	0	0.003	0.003
Bromus diandrus	0	0.005	0.032
Cakile maritima	0.217	0.024	0.004
Calystegia soldanella	0.027	0.026	0.009
Centaurea sphaerocephala	0	0.008	0.015
Chamaesyce peplis	0.097	0.014	0.001
Clematis flammula	0	0.004	0.018
Crucianella maritima	0	0.022	0.080
Cutandia maritima	0.008	0.036	0.095
Cyperus kalli	0.003	0.047	0.037
Daphne gnidium	0	0	0.001
Echinophora spinosa	0.029	0.029	0.004
Elymus farctus	0.161	0.134	0.044
Eryngium maritimum	0.021	0.020	0.001
Euphorbia terracina	0	0.003	0.028
Helicrisum stoechas	0	0.004	0.029
Juniperus oxycedrus	0	0	0.008
Lagurus ovatus	0	0.003	0.022
Lonicera implexa	0	0.001	0.002
Lophocloa pubescens	0	0.005	0.006
Lotus cytisoides	0	0.005	0.062
Medicago littoralis	0	0.021	0.081
Medicago marina	0.003	0.047	0.012
Ononis variegata	0.005	0.070	0.025
Otanthus maritimus	0.043	0.005	0
Pancratium maritimum	0.016	0.042	0.039
Phillirea angustifolia	0	0.002	0.005
Pistacia lentiscus	0	0.001	0.008
Plantago coronopus	0.003	0.013	0.017
Polygonum maritimum	0.038	0.003	0.001
Prasium majus	0	0.001	0.003
Pseudorlaya pumila	0	0.007	0.008
Pycnocomon rutifolium	0	0.003	0.042
Quercus ilex	0	0	0.003
Rubia peregrina	0	0	0.003
Salsola kali	0.193	0.025	0.002
Silene canescens	0	0.058	0.089
Smilax aspera	0	0.003	0.009
Sonchus bulbosus	0	0.006	0.005
Sporobolus virginicus	0.113	0.044	0.021
Vulpia fasciculata	0	0.009	0.073

References

1. Chiu C-H, Jost L, Chao A (2014) Phylogenetic beta diversity, similarity, and differentiation measures based on Hill numbers. Ecol Monogr 84: 21–44.
2. Leinster T, Cobbold CA (2012) Measuring diversity: the importance of species similarity. Ecology 93: 477–489.
3. Reeve R, Matthews L, Cobbold CA, Leinster T, Thompson J, Brummitt N. (2014) How to partition diversity. Cornell University Library, arXiv:1404.6520, assessed on May 28, 2014
4. Faith DP (1992) Conservation evaluation and phylogenetic diversity. Biol Conserv 61: 1–10.
5. Chao A, Chiu C-H, Jost L (2010) Phylogenetic diversity measures based on Hill numbers. Phil Trans R Soc B 365: 3599–3609.
6. Scheiner SM (2012) A metric of biodiversity that integrates abundance, phylogeny, and function. Oikos 121: 1191–1202.
7. Allen B, Kon M, Bar-Yam Y (2009) A new phylogenetic diversity measure generalizing the Shannon index and its application to phyllostomid bats. Am Nat 174: 236–243.
8. Guiasu RC, Guiasu S (2011) The weighted quadratic index of biodiversity for pairs of species: a generalization of Rao's index. Nat Sci 3:795–801.
9. Guiasu RC, Guiasu S (2012) The weighted Gini-Simpson index: revitalizing an old index of biodiversity. International J Ecol 2012, Article ID 478728, 10 pages.
