# Supplementary material for: Correction: Distance-Based Functional Diversity Measures and Their Decomposition: A Framework Based on Hill Numbers
Source: PLoS One. 2014 Nov 14;9(11):e113561. doi: 10.1371/journal.pone.0113561 (PMC4232602; doi:10.1371/journal.pone.0113561)
Supplement: Appendix S1 — Some properties of the proposed functional diversity measures. (PDF) [file pone.0113561.s001.pdf]

Supporting Information

Distance-based functional diversity measures and their decomposition: a framework based on Hill numbers

Chun-Huo Chiu and Anne Chao
Institute of Statistics, National Tsing Hua University, Hsin-Chu, Taiwan 30043

Appendix S1: Some properties of the proposed functional diversity measures

In this Appendix, we summarize some basic properties of the three classes of functional diversity measures: (1) functional Hill number ${}^qD(Q)$ which quantifies the effective number of equally abundant and equally distinct species in an assemblage with a constant species pairwise distance Q, where Q denotes Rao's quadratic entropy; (2) mean functional diversity ${}^qMD(Q)$ $=[{}^qD(Q)]\times Q$ which quantifies the effective sum of functional distances between a fixed species to all other species; and (3) (total) functional diversity ${}^qFD(Q)$ ($= {}^qD(Q)\times {}^qMD(Q)$), which quantifies the effective total distance between species of an assemblage. Since the three measures are closely related, all the properties are shared.

Without loss of generality, we assume that all distances d_{ij} are integers for intuitively understanding our derivation. This is because the measure ${}^qD(Q)$ is independent of the scale of d_{ij} and the other two measures are proportional to ${}^qD(Q)$ and thus properties discussed in this Appendix are not affected. As discussed by Chao et al. [1], we could conceptually think of all species pairwise distances as forming a single assemblage of pairwise distances. In this assemblage of "distances", there are d_{ij} units of "distance" for i, j = 1, 2, …, S. Each of the d_{ij} units has relative abundance $p_i p_j / Q$. From the definition of Rao's quadratic entropy, the sum of all these relative abundances is unity, i.e.,

$$\sum_{i=1}^{S}\sum_{j=1}^{S} d_{ij}\left(\frac{p_i p_j}{Q}\right) = 1.$$

Then we can apply the concept of Hill numbers to this assemblage of "distances". Our proposed (total) functional diversity of order q, ${}^qFD(Q)$, is the Hill number of order q for this assemblage of "distances":

$$\left[\sum_{i=1}^{S}\sum_{j=1}^{S} d_{ij}\left(\frac{p_i p_j}{Q}\right)^q\right]^{1/(1-q)}.$$

The measure ${}^qFD(Q)$ quantifies the effective total distances between species in the assemblage. From this perspective, the following properties are direct consequences of the theoretical properties of Hill numbers; see the appendices of [1] for details.

Proposition S1.1: The three measures ($^qD(Q)$, $^qMD(Q)$ and $^qFD(Q)$) are Schur-concave with respect to the product of relative abundances p_ip_j, and all three measures are non-increasing function of the order q.

Proposition S1.2: (Weak monotonicity) If a rarest new species is added to an assemblage, then the measure $^qFD(Q)$ does not decrease. Also, if a rarest new species is added to an assemblage such that the quadratic entropy remains unchanged, then all three measures do not decrease regardless of distance matrices.

Replication principle

Proposition S1.3: (Replication principle for functional Hill numbers) Suppose we have N equally large and completely distinct assemblages (no shares species). Assume that the functional Hill number $^qD(Q_{km})$ between Assemblage k and Assemblage m is a constant ^qD, for k, m = 1, 2, …, N. If these N assemblages are pooled, then the functional Hill number of order q in the pooled assemblage is $N \times {}^qD$.

Proof: Assume and there are S species in the pooled assemblage and the $S \times S$ symmetric species pairwise distance matrix is denoted by $[d_{ij}]$, i, j = 1, 2, …, S. Let $p_{ik} \geq 0$ be the relative abundance of ith species in the kth assemblage, i = 1, 2, …, S, k = 1, 2, …, N. The assumption that the functional Hill number of order q for any pair of assemblages is a constant $^q\mathcal{D}$ means

$$\sum_{i=1}^{S}\sum_{j=1}^{S}\frac{d_{ij}}{Q_{km}}(p_{ik}p_{jm})^q = ({}^qD)^{2(1-q)}, \quad k, m = 1, 2, \ldots, N, \qquad \text{(A1)}$$

where $Q_{km} = \sum_{i=1}^{S}\sum_{j=1}^{S} d_{ij}p_{ik}p_{jm}$ denotes the (abundance-weighted) mean distance between species of Assemblage k and Assemblage m. Eq. (A1) implies

$$\sum_{i=1}^{S}\sum_{j=1}^{S} d_{ij}(p_{ik}p_{jm})^q = Q_{km} \times ({}^qD)^{2(1-q)}, \quad k, m = 1, 2, \ldots, N. \qquad \text{(A2)}$$

When these N equally large assemblages are combined, the relative abundance of the ith species in the pooled assemblage is $\bar{p}_{i+} = \sum_{k=1}^{N} p_{ik}/N$, i = 1, 2,…, S. Then the quadratic entropy Q in the pooled assemblage is

$$Q = \sum_{i,j=1}^{S} d_{ij}\bar{p}_{i+}\bar{p}_{j+} = \sum_{i,j=1}^{S} d_{ij}\left(\sum_{k=1}^{N}\frac{p_{ik}}{N}\right)\left(\sum_{m=1}^{N}\frac{p_{jm}}{N}\right) = \frac{1}{N^2}\sum_{k=1}^{N}\sum_{m=1}^{N}\sum_{i,j=1}^{S} d_{ij}p_{ik}p_{jm} = \frac{1}{N^2}\sum_{k=1}^{N}\sum_{m=1}^{N} Q_{km}. \qquad \text{(A3)}$$

Therefore, the functional Hill number of order q in the pooled assemblage using Eq. 3 of the main text can be expressed as

$${}^qD_{\text{pooled}}(Q) = \left[\sum_{i=1}^{S}\sum_{j=1}^{S}\frac{d_{ij}}{Q}(\bar{p}_{i+}\bar{p}_{j+})^q\right]^{\frac{1}{2(1-q)}} = \left[\sum_{i=1}^{S}\sum_{j=1}^{S}\frac{d_{ij}}{Q}\left(\sum_{k=1}^{N}\frac{p_{ik}}{N}\right)^q\left(\sum_{m=1}^{N}\frac{p_{jm}}{N}\right)^q\right]^{\frac{1}{2(1-q)}}$$

$$= \left[\frac{1}{N^{2q}} \sum_{k,m=1}^{N} \sum_{i,j=1}^{S} \frac{d_{ij}}{Q} (p_{ik} p_{jm})^q \right]^{\frac{1}{2(1-q)}}.$$

It then follows from Eqs. (A2) and (A3) that we have

$${}^qD_{\text{pooled}}(Q) = \left[\frac{1}{N^{2q}} \sum_{k=1}^{N} \sum_{m=1}^{N} \frac{Q_{km}}{Q} ({}^qD)^{2(1-q)} \right]^{\frac{1}{2(1-q)}} = \left[\frac{1}{N^{2q}} N^2 ({}^qD)^{2(1-q)} \right]^{\frac{1}{2(1-q)}} = N \times {}^qD.$$

Thus, the functional Hill number of the same order q in the pooled assemblage is N times that of each individual assemblage. Notice that in our proof of this replication principle, the mean distances Q_{km}, k, m = 1, 2, …, N, are not required to be a constant. Also, the species abundance distributions are allowed to be different across assemblages, and the result is valid for any symmetric matrices. This is a strong version of the replication principle.

If we further assume that all the mean distances Q_{km}, k, m = 1, 2, …, N, are the same, then the mean functional diversity of the pooled assemblage will be N times that of each assemblage. However, the total functional diversity (as a product of functional Hill number and the mean functional diversity) satisfies a "*quadratic* replication principle", i.e., the total functional diversity of the pooled assemblage will be N^2 times that of each assemblage. We only state the propositions below and the proof follows directly from Proposition S1.3.

Proposition S1.4: (Replication principle for the mean functional diversity ${}^qMD(Q)$) Suppose we have N equally large and completely distinct assemblages (no shares species). Assume that for all pairs of assemblages (k, m) the mean functional diversity ${}^qMD(Q_{km})$ for k, m = 1, 2, …, N are identical, and the mean distances Q_{km} for k, m = 1, 2, …, N are also identical. When these N equally large assemblages are pooled, the mean functional diversity of the pooled assemblage is N times that of an individual assemblage.

Proposition S1.5: (Quadratic replication principle for the total functional diversity ${}^qFD(Q)$) Suppose we have N equally large and completely distinct assemblages (no shares species). Assume that for all pairs of assemblages (k, m) the total functional diversity ${}^qFD(Q_{km})$ for k, m = 1, 2, …, N are identical, and the mean distances Q_{km} for k, m = 1, 2, …, N are also identical. When these assemblages are pooled, the total functional diversity of the pooled assemblage is N^2 times that of an individual assemblage.

Reference

1. Chao A, Chiu C-H, Jost L (2010) Phylogenetic diversity measures based on Hill numbers. Phil Trans R Soc B 365: 3599–3609.
