# Supplementary material for: Correction: Distance-Based Functional Diversity Measures and Their Decomposition: A Framework Based on Hill Numbers
Source: PLoS One. 2014 Nov 14;9(11):e113561. doi: 10.1371/journal.pone.0113561 (PMC4232602; doi:10.1371/journal.pone.0113561)
Supplement: Appendix S4 — Functional beta diversity and functional diversity excess lead to the same classes of similarity and differentiation measures. (PDF) [file pone.0113561.s004.pdf]

Supporting Information

Distance-based functional diversity measures and their decomposition: a framework based on Hill numbers

Chun-Huo Chiu and Anne Chao
Institute of Statistics, National Tsing Hua University, Hsin-Chu, Taiwan 30043

Appendix S4: Functional beta diversity and functional diversity excess lead to the same classes of similarity and differentiation measures

As indicated in the main text, we can apply the additive decomposition to the three measures, ${}^qD(Q)$ (Eq. 3), ${}^qMD(Q)$ (Eq. 4a) and ${}^qFD(Q)$ (Eq. 4b); here the equations numbers refer to those in the main text. For example, we define the "functional diversity excess" as ${}^qFD_\gamma(Q) - {}^qFD_\alpha(Q)$. This is also an interpretable measure. However, this excess measure cannot be directly applied to compare the functional similarity or differentiation among assemblages across multiple sets of assemblages because it depends not only on the number of assemblages, but also on the corresponding functional alpha (equivalently, gamma) diversity. Following Chao et al. [1] and Chiu et al. [2], we can readily eliminate these dependences by using an appropriate normalization. For easy demonstration, we prove the theorem for differentiation measures instead of similarity measures.

(1) *A class of functional distance-differentiation measures from a local perspective*: $1 - C^*_{qN}(Q)$

We first prove the following two inequalities:

$$0 \le [{}^qFD_\alpha(Q)]^{1-q} - [{}^qFD_\gamma(Q)]^{1-q} \le (1 - N^{2(1-q)})[{}^qFD_\alpha(Q)]^{1-q}, \; q > 1, \qquad \text{(D1)}$$

and

$$0 \le [{}^qFD_\gamma(Q)]^{1-q} - [{}^qFD_\alpha(Q)]^{1-q} \le (N^{2(1-q)} - 1)[{}^qFD_\alpha(Q)]^{1-q}, \quad 0 \le q < 1. \qquad \text{(D2)}$$

It follows from ${}^qFD_\gamma(Q) \le N^2[{}^qFD_\alpha(Q)]$ (Proposition S2.1 of Appendix S2) that for $q > 1$, we have $[{}^qFD_\gamma(Q)]^{1-q} \ge N^{2(1-q)}[{}^qFD_\alpha(Q)]^{1-q}$. Then we obtain the inequality (D1). For $0 \le q < 1$, the inequality (D2) also follows directly from the same inequality. Dividing $[{}^qFD_\alpha(Q)]^{1-q} - [{}^qFD_\gamma(Q)]^{1-q}$ (for $q > 1$) and $[{}^qFD_\gamma(Q)]^{1-q} - [{}^qFD_\alpha(Q)]^{1-q}$ (for $0 \le q < 1$) by their respective maximum possible value gives the measure $1 - C^*_{qN}(Q)$. That is,

$$\frac{[{}^qFD_\gamma(Q)]^{1-q} - [{}^qFD_\alpha(Q)]^{1-q}}{(N^{2(1-q)} - 1)[{}^qFD_\alpha(Q)]^{1-q}} = \frac{[{}^qFD_\gamma(Q)/{}^qFD_\alpha(Q)]^{1-q} - 1}{N^{2(1-q)} - 1} = \frac{[{}^qFD_\beta(Q)]^{1-q} - 1}{N^{2(1-q)} - 1} = 1 - C^*_{qN}(Q).$$

Also, for $q = 1$, we have

$$\lim_{q\to 1}\frac{[{}^qFD_\gamma(Q)]^{1-q}-[{}^qFD_\alpha(Q)]^{1-q}}{(N^{2(1-q)}-1)[{}^qFD_\alpha(Q)]^{1-q}}=\frac{\log{}^1FD_\gamma(Q)-\log{}^1FD_\alpha(Q)}{2\log N}=1-C^*_{1N}(Q).$$

(2) *A class of functional distance-differentiation measures from a regional perspective*: $1-U^*_{qN}(Q)$

The inequality in Proposition S2.1 of Appendix S2, ${}^qFD_\gamma(Q)/N^2 \le {}^qFD_\alpha(Q) \le {}^qFD_\gamma(Q)$, implies $(1/N^{2(1-q)})[{}^qFD_\gamma(Q)]^{1-q} \le [{}^qFD_\alpha(Q)]^{1-q} \le [{}^qFD_\gamma(Q)]^{1-q}$ for $0 \le q < 1$. Thus, we obtain

$$0 \le [{}^qFD_\gamma(Q)]^{1-q}-[{}^qFD_\alpha(Q)]^{1-q} \le (1-1/N^{2(1-q)})[{}^qFD_\gamma(Q)]^{1-q},\ \ 0 \le q < 1;$$

and

$$0 \le [{}^qFD_\alpha(Q)]^{1-q}-[{}^qFD_\gamma(Q)]^{1-q} \le (1/N^{2(1-q)}-1)[{}^qFD_\gamma(Q)]^{1-q},\ \ q > 1.$$

Dividing $[{}^qFD_\gamma(Q)]^{1-q}-[{}^qFD_\alpha(Q)]^{1-q}$ (for $0 \le q < 1$) and $[{}^qFD_\alpha(Q)]^{1-q}-[{}^qFD_\gamma(Q)]^{1-q}$ (for q >1) by their respective maximum possible value gives the measure, $1-U^*_{qN}(Q)$. That is,

$$\frac{[{}^qFD_\gamma(Q)]^{1-q}-[{}^qFD_\alpha(Q)]^{1-q}}{(1-1/N^{2(1-q)})[{}^qFD_\gamma(Q)]^{1-q}}=\frac{1-[{}^qFD_\alpha(Q)/{}^qFD_\gamma(Q)]^{1-q}}{1-1/N^{2(1-q)}}=\frac{1-[1/{}^qFD_\beta(Q)]^{1-q}}{1-1/N^{2(1-q)}}=1-U^*_{qN}(Q).$$

Moreover, we have

$$\lim_{q\to 1}\frac{[{}^qFD_\gamma(Q)]^{1-q}-[{}^qFD_\alpha(Q)]^{1-q}}{(1-1/N^{2(1-q)})[{}^qFD_\gamma(Q)]^{1-q}}=\frac{\log{}^1FD_\gamma(Q)-\log{}^1FD_\alpha(Q)}{2\log N}=1-U^*_{1N}(Q)=1-C^*_{1N}(Q).$$

(3) *A class of measures of functional distance-heterogeneity*: $1-S^*_{qN}(Q)$

As we proved in Appendix S2, $[{}^qFD_\gamma(Q)]/N^2 \le {}^qFD_\alpha(Q) \le {}^qFD_\gamma(Q)$. We then have

$$0 \le {}^qFD_\gamma(Q)]-{}^qFD_\alpha(Q) \le (1-1/N^2)[{}^qFD_\gamma(Q)],$$

which shows the functional diversity excess depends on functional gamma diversity and *N*. The normalized measure turns out to be $1-S^*_{qN}(Q)$:

$$[{}^qFD_\gamma(Q)-{}^qFD_\alpha(Q)]/\{(1-1/N^2)[{}^qFD_\gamma(Q)]\}=[1-1/{}^qFD_\beta(Q)]/(1-1/N^2)=1-S^*_{qN}(Q).$$

(4) *A class of functional distance-turnover rate*: $1-V^*_{qN}(Q)$

As proved in Appendix S2, we have ${}^qFD_\alpha(Q) \le {}^qFD_\gamma(Q) \le N^2[{}^qFD_\alpha(Q)]$. This implies

$$0 \le {}^qFD_\gamma(Q)-{}^qFD_\alpha(Q) \le (N^2-1)[{}^qFD_\alpha(Q)],$$

which shows the functional diversity excess depends on the functional alpha diversity and *N*. The dependence can be removed in this case by normalization and the resulting measure is $1-V^*_{qN}(Q)$:

$$[{}^{q}FD_{\gamma}(Q) - {}^{q}FD_{\alpha}(Q)] / \{(N^2 - 1)[{}^{q}FD_{\alpha}(Q)]\} = [{}^{q}FD_{\beta}(Q) - 1] / (N^2 - 1) = 1 - V^*_{qN}(Q) .$$

Thus, the functional diversity excess leads to the same four classes of normalized similarity measures ($C^*_{qN}(Q)$, $U^*_{qN}(Q)$, $V^*_{qN}(Q)$ and $S^*_{qN}(Q)$) derived from the functional beta diversity.- Although normalization is not a general cure for problems of dependence on alpha or gamma functional diversity, all the resulting normalized measures discussed above are in terms of functional beta diversity only. Thus, dependence on the alpha (or gamma) functional diversity can be removed in our cases as our functional beta diversity is independent of the functional alpha diversity and also independent of the functional gamma diversity, based on a similar argument in [1].

References

1. Chao A, Chiu C-H, Hsieh TC (2012) Proposing a resolution to debates on diversity partitioning. Ecology 93: 2037–2051.
2. Chiu C-H, Jost L, Chao A (2014) Phylogenetic beta diversity, similarity, and differentiation measures based on Hill numbers. Ecol Monogr 84: 21–44.
